# p53 is functionally inhibited in clear cell renal cell carcinoma (ccRCC): a mechanistic and correlative investigation into genetic and molecular characteristics

**DOI:** 10.1007/s00432-021-03786-1

**Published:** 2021-09-09

**Authors:** Karoline Diesing, Silvia Ribback, Stefan Winter, Manuela Gellert, Antonia M. Oster, Viktoria Stühler, Eva Gläser, Frank Adler, Christoph Hartwig, Markus Scharpf, Jens Bedke, Martin Burchardt, Matthias Schwab, Christopher H. Lillig, Nils Kroeger

**Affiliations:** 1 Department of Urology, University Medicine Greifswald, Ferdinand-Sauerbruch-Straße, E17475 Greifswald, Germany; 2 The Institute of Medical Biochemistry and Molecular Biology, University Medicine Greifswald, Greifswald, Germany; 3 The Dr. Margarete Fischer-Bosch Institute of Clinical Pharmacology, Stuttgart, Germany; 4 The University of Tübingen, Tübingen, Germany; 5 The Institute of Pathology, University Medicine Greifswald, Greifswald, Germany; 6 The Institute of Radiation Oncology, University Medicine Greifswald, Greifswald, Germany; 7 The Institute of Pathology, University of Tübingen, Tübingen, Germany; 8 The Department of Urology, University of Tübingen, Tübingen, Germany; 9 Departments of Clinical Pharmacology, Pharmacy and Biochemistry, University of Tübingen, Tübingen, Germany

**Keywords:** p53, Tumor suppressor gene, Biomarker, Renal cell carcinoma, The Cancer Genome Atlas

## Abstract

**Purpose:**

Although p53 is rarely mutated in ccRCC, its overexpression has been linked to poor prognosis. The current study sought to elucidate the unique role of p53 in ccRCC with genomic, proteomic, and functional analyses.

**Materials and methods:**

Data from the Cancer Genome Atlas (TCGA) were evaluated for genomic and proteomic characteristics of p53; a tissue micro array (TMA) study was carried out to evaluate the association of p53 and phosphorylated p53 (pp53) with clinical outcome. Mechanistic in vitro experiments were performed to confirm a pro-apoptotic loss of p53 in ccRCC and p53 isoforms as well as posttranslational modifications of p53 where assessed to provide possible reasons for a functional inhibition of p53 in ccRCC.

**Results:**

A low somatic mutation rate of p53 could be confirmed. Although mRNA levels were correlated with poor prognosis and clinicopathological features, there was no monotonous association of mRNA levels with survival outcome. Higher p53 protein levels could be confirmed as poor prognostic features. In vitro, irradiation of ccRCC cell lines markedly induced levels of p53 and of activated (phosphorylated) p53. However, irradiated ccRCC cells demonstrated similar proliferation, migration, and p53 transcriptional activity like non-irradiated controls indicating a functional inhibition of p53. p53 isoforms and could not be correlated with clinical outcome of ccRCC patients.

**Conclusions:**

p53 is rarely mutated but the wildtype p53 is functionally inhibited in ccRCC. To investigate mechanisms that underlie functional inhibition of p53 may provide attractive therapeutic targets in ccRCC.

**Supplementary Information:**

The online version contains supplementary material available at 10.1007/s00432-021-03786-1.

## Introduction

The transcription factor p53 is one of the most often investigated tumor suppressor genes in human cancer. It has been demonstrated that p53 is able to induce context dependent either cell cycle arrest, apoptosis, or senescence and many other biological processes that inhibit carcinogenesis and cancer progression (reviewed in Kastenhuber and Lowe 2017). p53 is often accumulated in advanced ccRCC stages and accumulation of p53 has been linked to a shorter cancer-specific- (CSS) and recurrence-free-survival (PFI) (Noon et al. 2010; Shvarts et al. 2005). In consequence, it is a molecular poor prognostic factor (Klatte et al. 2009).

Accumulation of p53 has been explained by p53 mutations that inhibit its degradation and thus, leads to more frequent p53 staining in ccRCC (Noon et al. 2010; Shvarts et al. 2005). However, this hypothesis has been challenged in some studies. Noon et al. (2011) have validated p53 as a poor prognostic factor in ccRCC but p53 mutations were only present in ~ 2% of their whole study cohort and 86% of the tumors with p53 overexpression retained wild-type (wt) p53. This study was performed in a study cohort with modest sample size.

Therefore, in the current study it was hypothesized that p53 *wt* accumulates and is associated with advanced tumor stages and poor prognosis in ccRCC. For this purpose, data of the Cancer Genome Atlas Project (TCGA), a tissue micro array (TMA) analysis and mechanistic *in-vitro* experiments were conducted to get a comprehensive view about p53 and its activated form phosphorylated p53 in ccRCC. Furthermore, p53 transcript variants were investigated to provide possible explanation why p53 *wt* may be functionally inhibited in ccRCC.

## Methods and materials

### Patient and tumor characteristics

In the current study, three patient cohorts were analyzed: a cohort of the Cancer Genome Atlas project (suppl. Table 1), the Department of Urology at the University Hospital in Tübingen/Germany (Table 2) and from the University Medicine Department of Urology in Greifswald/Germany (suppl. Table 2).

### Tissue micro array (TMA) analyses

We analyzed staining frequencies and combined the frequency and intensity measures into an integrated intensity measure using the following formula: ((% staining at intensity 3*3) + (% staining at intensity 2*2) + (% staining at intensity 1*1))/100 as described previously (Seligson et al. 2013). A list of the antibodies and anti-body dilutions and details of TMA construction are provided in the supplements.

#### In-vitro experiments

The detailed description of all in-vitro experiments is demonstrated in the supplements.

### Statistical analyses and outcomes

#### Analysis of genetic alterations in the TCGA

The primary endpoint was the current TCGA analyses was disease specific survival (DSS). For explanatory reasons overall (OS) and progression free interval (PFI) are also shown in the supplements. Associations between clinicopathological variables and copy number variations (CNV) or somatic mutations were investigated using Fisher’s exact test, Cochran-Armitage trend test or Mann–Whitney *U* test as appropriate. Uni- and multivariable Cox proportional hazard (PH) regression were applied for association analyses between copy number variations (CNV) or somatic mutations and DSS Here survival data was obtained from Liu et al*. *(2018). The analyzed TCGA data consists of tumor samples of 459 patients (suppl. Table 1). In the different subsets (somatic mutation analyses, RNAseq data, CNV) the numbers of available patient data may differ. For example, for somatic mutations are only data of 290 patients available in the TCGA data.

#### Statistical analyses of the tissue microarray and PCR studies

p53 protein and mRNA-expression data were correlated with CSS, clinicopathological features. Descriptive statistics included continuous variables that are shown as mean ± standard deviation (SD) or interquartile ranges (IQR) whereas categorical data are shown as absolute numbers and corresponding frequencies. All categorical comparisons of the TMA analysis have been tested with Fisher’s exact test.

Survival functions were estimated with the Kaplan–Meier method and associations with survival times were assessed with uni- and multivariable Cox PH regression analyses. Restricted cubic splines were only used for the analyses of the RNA Seq data to demonstrate to nonlinear monotony. For this purpose, we have tested models with three, four and five knots. The model with the smallest Akaike information criteria (AIC) was chosen. OS was calculated from the date of surgery to any reason of death or last contact. PFI was the period from surgery to development of distant metastases or local recurrence.

All statistical test were two-sided and statistical significance was defined as *p*<0.05. All data were analyzed with the Statistical Package for Social Sciences software, version 24.0 (SPSS Inc., Chicago, IL) and R version Rx64 3.5.0 (https://www.r-project.org/), including additional packages coin, survival and rms.

## Results

### Aspects on p53 from the TCGA

The clinicopathological characteristics of the analyzed patients and their tumor in the TCGA are shown in suppl. Table 1. The TCGA data were investigated for somatic mutations of the p53 gene (TP53) in ccRCC. Only 5/290 (1.7%) of the samples had somatic mutations. Mutations could be found over all tumor stages without a clear association with advanced tumor stages (suppl. Figure 1). However, patients with p53 somatic mutations had a significant worse CSS (*p*<0.001 (suppl. Figure 2).

Next, the associations of mRNA expression levels of TP53 and clinicopathological features were analyzed. TP53 mRNA levels were higher in stage III and stage IV ccRCC (*p*=0.0024). Moreover, TP53 mRNA was higher expressed in patients with distant metastases, lymph node metastases, higher T stages or higher histological grades (Suppl. Figure 3). However, the analyses of the survival endpoints demonstrated no monotone association of TP53 mRNA levels with CSS: patients with mediumTP53 mRNA expression showed better survival than patients with low or high expression levels (Suppl. Figure 4).

Additionally, the protein data of p53 in the TCGA were analyzed. Higher p53 protein levels were associated with worse survival outcome in univariate analysis (HR; 95% CI; *p* value (logrank test): DSS 2.41 (1.13–5.13) 0.024; suppl. Figure 5). After adjustment for TNM stages and grading p53 Protein levels could not be confirmed as independent prognostic factor in ccRCC (HR; 95% CI; *p *value (logrank test: DSS 1.92 (0.86–4.27) 0.111.

**Fig. 1:**
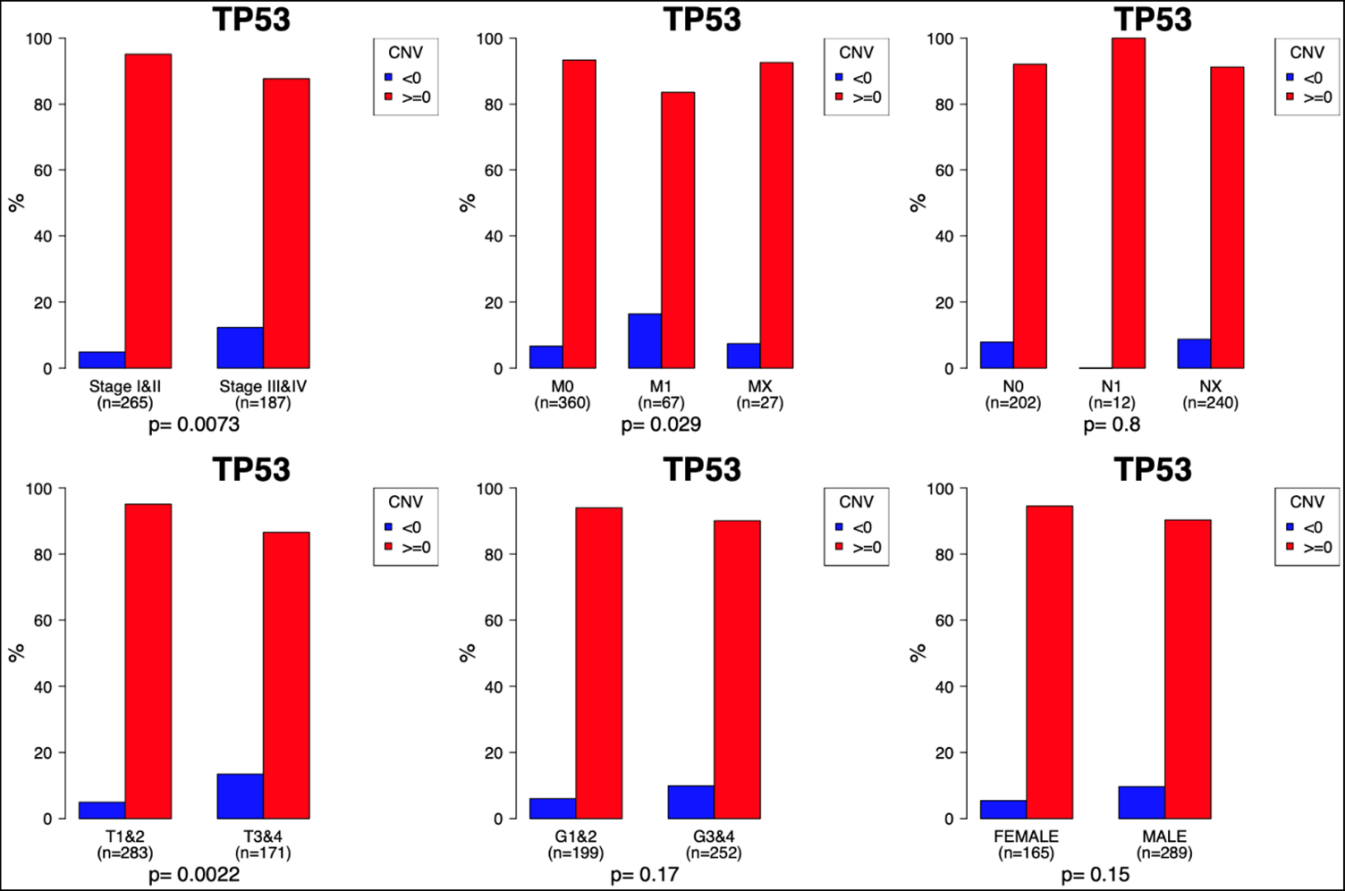
Comparison of clinicopathological features according to copy number variations (CNV). Demonstrates the comparisons in copy number variations (CNV). Values < 0 represent homozygous deletions (−2), single copy deletions (−1) and values≥0 diploid normal copy (0), low-level amplification (1), and high-level amplifications (2)

Copy number variations (CNV) in the TP53 gene were observed only in a minority of the TCGA ccRCCs. There were 53/454 (11.7%) tumors that had CNV. Deletions could be found in all tumor stages (Fig. 1). Interestingly, amplifications were more often found in low than in high tumor stages (Stage I+II vs. III+IV *p*=0.0073; M0 vs. M1 *p*=0.013; T1+T2 vs. T3+T4 *p*=0.002; N0 vs. N1 *p*=0.61; G1+G2 vs. G3+G4 *p*=0.17; Fig. 1).

Expression data of twelve TP53 exon positions were available on Xena (https://xenabrowser.net). Three exon positions (chr17: 7565097–7565332, chr17: 7576525–7576657: chr17: 7580643–7580745) showed low expression for all samples (RPKM<3) and were therefore not considered in the subsequent analyses. The evaluation of the expression levels of the remaining nine positions demonstrated that all positions had significant higher expression levels in tumor than in adjacent normal kidney tissue. Survival analysis demonstrated that for eight of the nine exons investigated, increased expression was significantly associated with DSS (*p* values < 0.001; suppl. Figures 6–8). In contrast, increased expression of chr17:7571720−7573008 was significantly associated with worse DSS (p-values p<0.001; suppl. Figure 6-8). The comparison of the clinicopathological features (UICC stages, TNM and Fuhrman grading) according to the expression of the nine exon positions demonstrated no significant associations for all CNV (analyses not shown).

In multivariable analyses, all p53 results were corrected for TNM stages and Fuhrmann grading. RNA seq data, p53 protein expression, and copy number variations did not show a significant association with cancer specific survival outcome in the TCGA cohort (Table 1). The expression of nine of twelve evaluable TP53 exons demonstrated an association for independent better DSS while chr17:7571720–7573008: was associated with worse DSS only in univariable analysis Table 1.

**Table 1:** Multivariable analyzes for the association of p53 with cancer specific survival outcome in the TCGA cohort

	HR	Lower limit of 95% CI	Upper limit of 95% CI	*p *value (Wald test)
RNA Seq TP53	1.25	0.72	2.18	0.428
Protein expression p53	1.75	0.74	4.14	0.204
Copy number variations				
TP53	1.06	0.59	1.91	0.833
Chromosome 17 Exone expression				
chr17:7571720–7573008	1.24	0.74	2.08	0.423
chr17:7573927–7574033	0.67	0.51	0.88	0.00384
chr17:7576853–7576926	0.69	0.52	0.9	0.00616
chr17:7577019–7577155	0.7	0.54	0.92	0.0103
chr17:7577499–7577608	0.73	0.57	0.94	0.0153
chr17:7578371–7578811	0.64	0.47	0.87	0.0039
chr17:7579312–7579590	0.72	0.57	0.91	0.00593
chr17:7579700–7579940	0.68	0.53	0.89	0.00396
chr17:7590695–7590863	0.72	0.55	0.95	0.021

In multivariable analyses, p53 results were corrected for TNM stages and Fuhrman grading. RNA seq data, p53 protein expression, and copy number variations did not show a significant association with cancer specific survival outcome in the TCGA cohort. The expression of nine of twelve evaluable TP53 exons demonstrated an association for independent better cancer specific survival while chr17:7571720–7573008 was associated with worse cancer specific survival only in univariable analysis

Lastly, also methylation data methylation data of cg sites in TP53 (± 2 kb) were analyzed. However, we were unable to demonstrate associations between methylation patterns cg sites in TP53 with gen- or protein expression. All correlation coefficients were < 0.2.

### TMA Validation of pp53 and p53 in ccRCC

To validate the prognostic relevance and to analyze the role of activated p53 in ccRCC, the expression of p53 and phosphorylated p53 (pp53) was examined in a TMA of 274 ccRCC patients. The clinicopathological characteristics of the analyzed patients and their tumor in the TMA are shown in suppl. Table 2. The median follow-up time was 89 months (IQR 25th–75th percentile 21–152 months) and 72/253 patients (26%) had died at the time of analysis. Fourteen ccRCCs were not evaluable due to loss of tumor spots during the antigen retrieval and staining process.

All comparisons are shown in Table 2. p53 and pp53 expression was found in only 33/242 (12%) and 98/242 (37%) evaluable ccRCCs, respectively. There was a statistical difference for the association of p53 expression with the frequency of lymph node metastases (4/33 (12) vs. (6/222 (3%), *p* = 0.026) and there was also a significant association of pp53 intensity x frequency with lymph node metastases. Otherwise, there was no difference in tumors with vs. without p53 expression. Likewise, there was no difference in ccRCCs with vs. without expression of phosphorylated (activated) p53 except for T stages (*p* = 0.044). These differences did not proof to be significant after correction for multiple comparisons. The univariable comparison of DSS of ccRCC patients with vs. without p53 or pp53 expression with Kaplan–Meier analyses revealed no statistically provable difference (p53 *p* = 0.943; pp53 *p* = 0.381) (suppl. Figure 9 A and B). Next, patients with non-metastatic and metastatic ccRCC (mccRCC) were analyzed separately for differences in DSS according to p53 or pp53 expression. There was no difference in DSS of patients with ccRCCs expressing pp53 vs. no pp53 (log rank *p* = 0.524) and there was also no statistically provable difference in DSS for tumors with p53 expression vs. no p53 expression (*p* = 0.102) in non-metastatic ccRCCs. Similarly, there was no statistical difference for both p53 (*p* = 0.316) and pp53 (*p* = 0.726) expression in mccRCC.

**Table 2:** Clinicopathological features in ccRCC tumor samples with p53 or phosphorylated p53 according to staining frequencies and intensities

Features	p53 Expression (intensity × frequency)			Phosphorylated p53 expression (intensity × frequency)		
	Mean	SEM	*p *value	Mean	SEM	*p *value
Gender						
Male	2.28	1.04	0.424	5.83	1.16	0.272
Female	1.35	0.52		8.77	2.40	
T stage						
Localized (T1 + T2)	1.65	0.66	0.610	6.82	1.46	0.878
Advanced (T3)	2.56	1.66		6.49	1.55	
N stage						
N0	1.30	0.43	0.283	6.94	1.12	<0.001
N+ (N1 + N2)	19.30	15.77		0.50	0.50	
M stage						
M 0	1.84	0.78	0.635	6.62	1.14	0.878
M 1	3.03	2.37		7.18	3.40	
Fuhrman grade						
Low grade (G1 and G2)	1.38	0.49	0.327	6.84	1.18	0.737
High Grade (G3 and G4)	5.49	4.11		5.85	2.71	
Tumor necrosis						
Yes	3.39	0.45	0.185	6.17	1.25	0.580
No	1.04	1.70		7.45	1.93	
Sarcomatoid						
Yes	2.35	1.36	0.808	8.71	5.99	0.728
No	1.97	0.79		6.55	1.08	

Lastly, the product of staining frequency and intensity of p53 and pp53 was used as a continuous variable to investigate the association with DSS. The combined p53 staining frequency and intensity (see methods and materials part 2.2) was associated with DSS in univariable analysis (HR 1.01, 95% CI 1.00–1.03, *p* = 0.019). There was no statistical association with DSS for pp53 staining intensity × frequency (*p* = 0.731). In multivariable analysis, p53 staining intensity × frequency lost its significance after adjusting for TNM stages and Fuhrman grading. Then conditional interference tree analyses were applied to calculate a possible cutoff value based on the score frequency × intensity (IntMax-Score). However, no systematic cutoff could be determined based on this systematic statistical approach for both pp53 and p53.

### In-vitro studies: functional loss of p53 in ccRCC

#### Irradiation induces p53 accumulation and activation in ccRCC cell lines

**Fig. 2:**
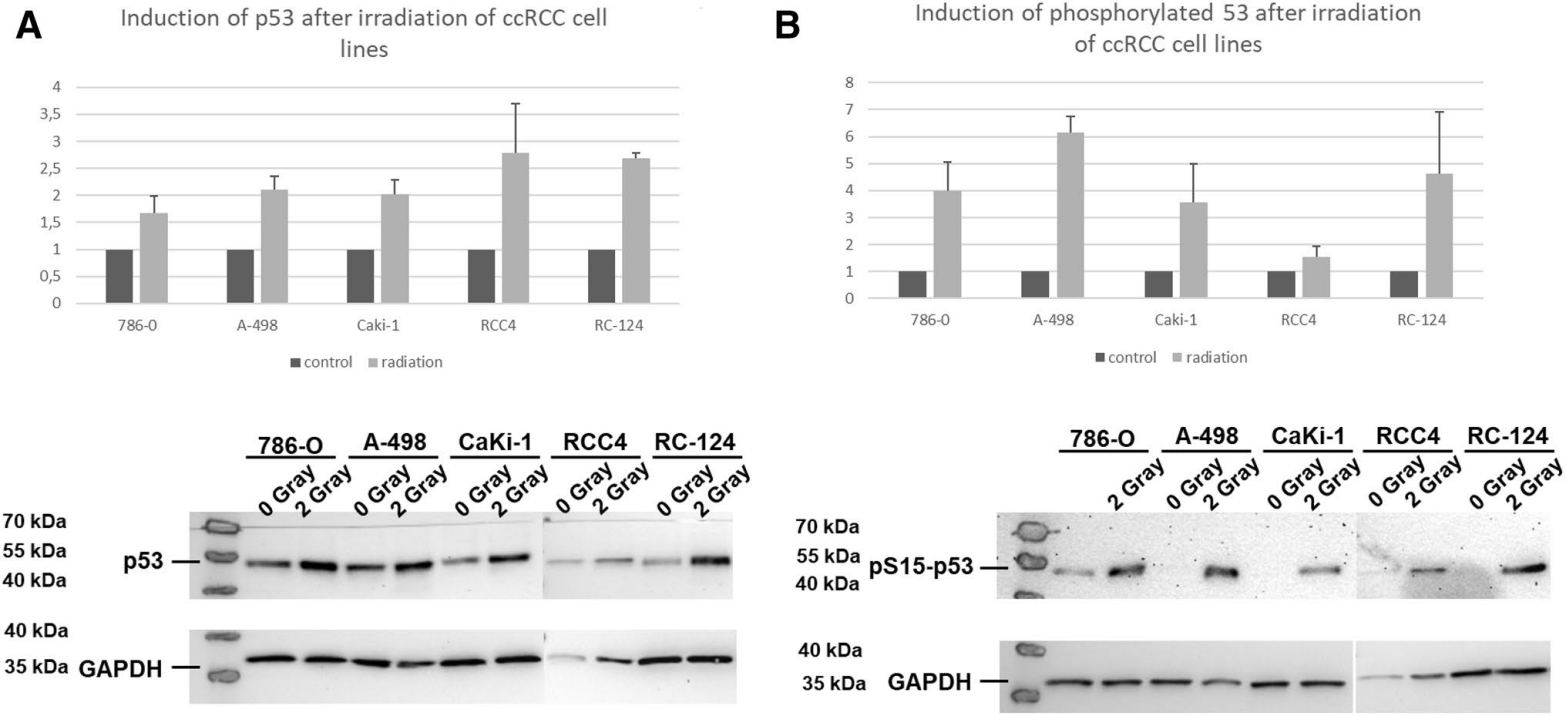
p53 and phosphorylated p53 after irradiation of ccRCC cell lines. Irradition of ccRCC cell lines demonstrated a significant induction of p53 levels and phosphorylated p53 levels. Error bars represent SEM

The ccRCC cell lines 786-0, Caki-1, RCC4, A-498 and the kidney cell line RC-124 were irradiated with 2 Gray. The protein content of p53 and pp53 was determined with western blot analyzes. As demonstrated in Fig. 2 irradiation induced a significant increase (1.67 fold (786–0)–2.78 fold (RCC4); Fig. 2A) of p53 levels in all cell lines. Following irradiation, p53 is activated by several posttranslational modifications including phosphorylation at serine 15 (pS15) (Lavin and Gueven 2006). Phosphorylation of p53 is an accepted sign of activation (Lavin and Gueven 2006). A considerable p53 phosphorylation (1.53 fold (RCC4)–6.16 fold (A-498) (Fig. 2B) was observed in all cell lines.

#### Migration and proliferation are similar after irradiation in irradiated ccRCC cell lines and controls

**Fig. 3:**
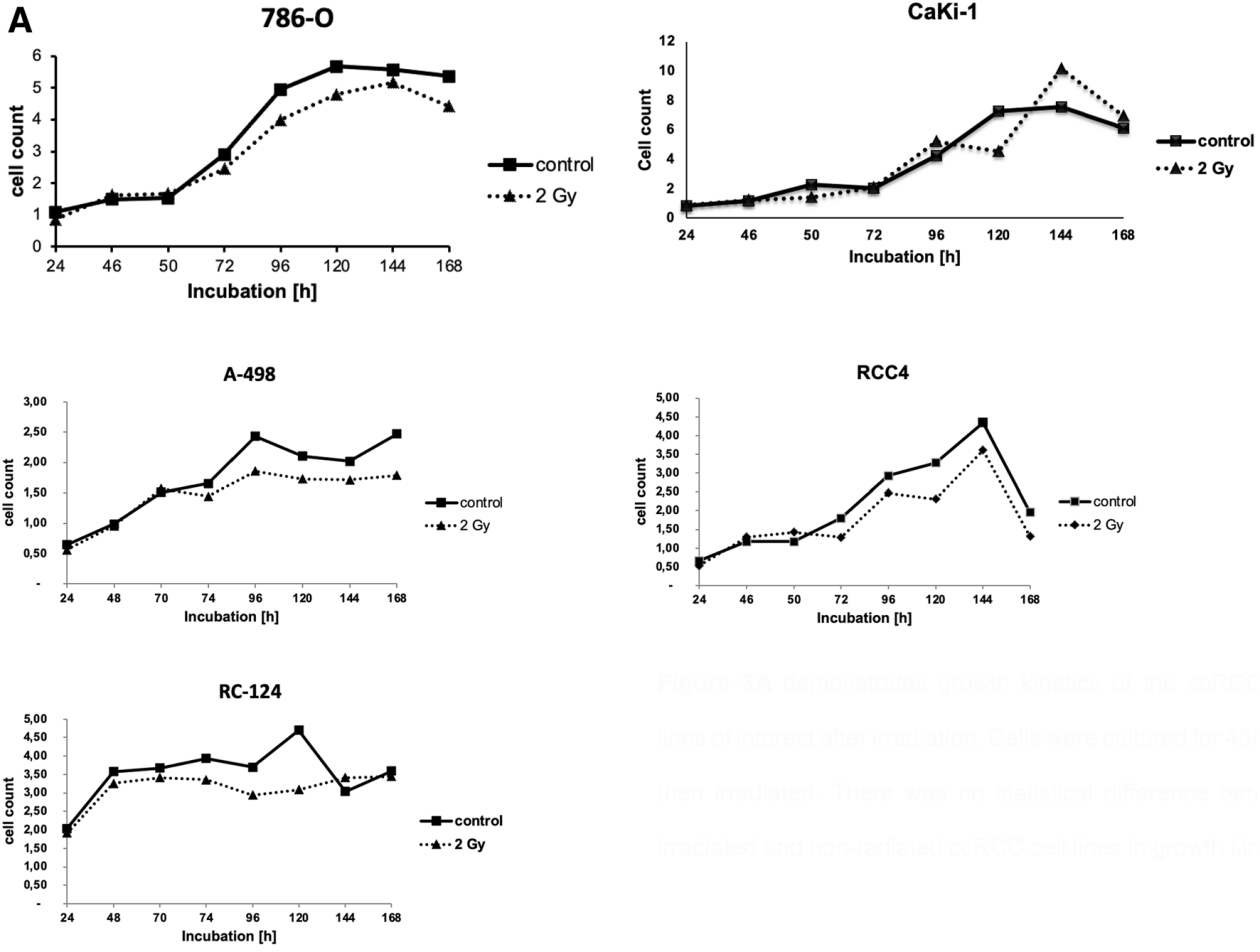

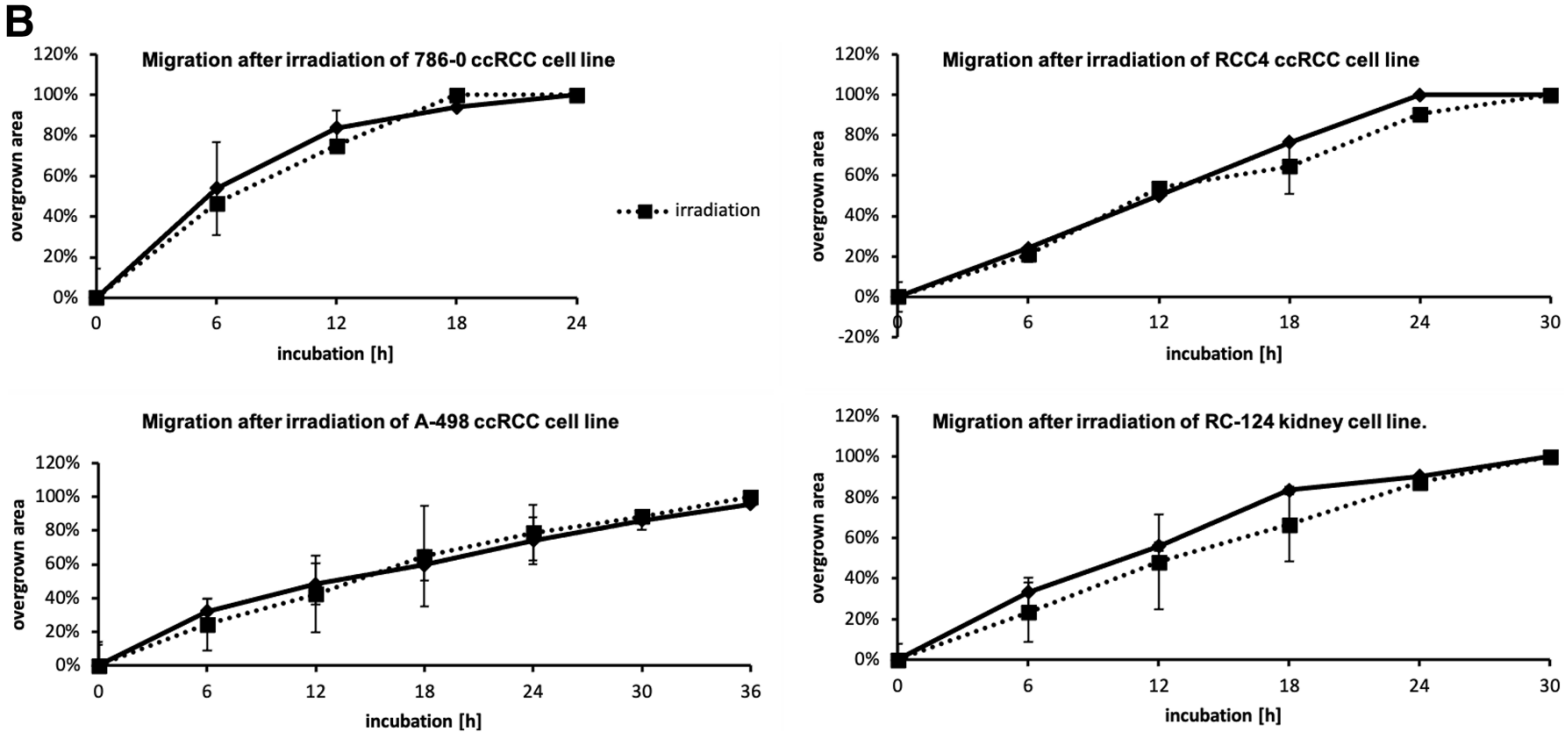
**A** Growth kinetics of the ccRCC cell lines of interest after irradiation. Cells were cultured for 48 h and then irradiated. There was no statistical difference between irradiated and non-radiated ccRCC cell lines in growth kinetics. **B** Migration in ccRCC cell lines 786–0, RCC4, A-498, and the kidney cell line RC-124

Furthermore, we examined migration and proliferation in ccRCC cell lines and the non-malignant cell line RC-124. No difference in proliferation could be observed after irradiation between RCC cell lines and control (Fig. 3A). Similarly, after irradiation there were no differences in migration of all ccRCC cell lines in comparison to controls (Fig. 3B).

#### Transcriptional activity of p53 after irradiation in ccRCC cell lines and controls

**Fig. 4:**
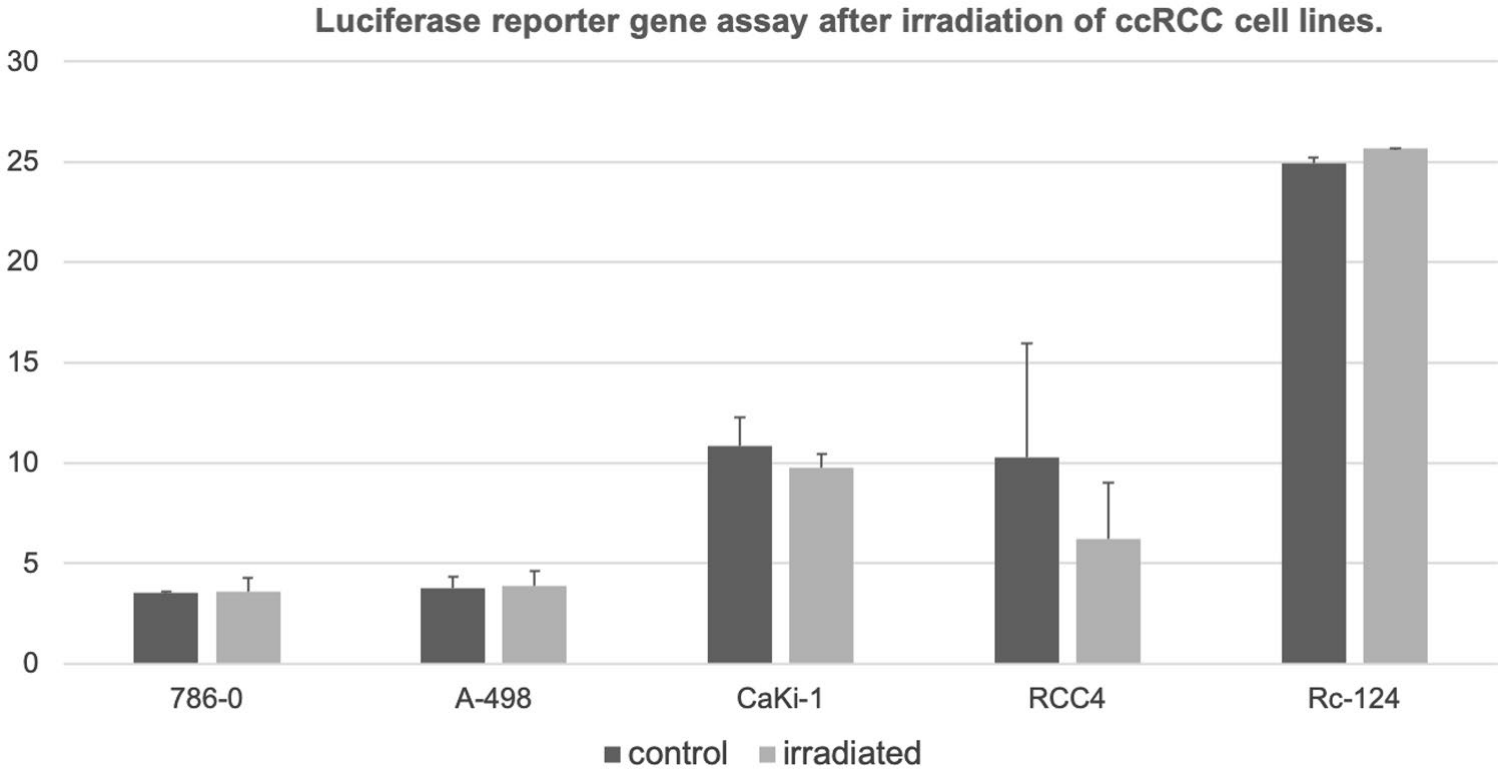
Results of a transfection with a dual firefly luciferase assay. There was less transcriptional activity in the cell line RCC4. Otherwise, no specific activation of p53 transcriptional activity could be recognized

Next, a reporter gene assay was applied to examine p53 transcriptional activity. No difference in p53 transcriptional activity could be detected as demonstrated in Fig. 4.

### Isoforms of p53 in clinical specimens of ccRCC

Next, the levels of p53 isoforms were investigated in cancer and normal tissue specimens of 55 ccRCC patients. The isoforms ∆40 α and ∆40 γ could not be detected in all ccRCC tumor samples of this patient cohort.

The comparison of clinicopathological characteristics is demonstrated is demonstrated in Table 3. In summary, the only significant result after α-error (*p* = 0.05) Bonferroni correction (*p* = 0.05/45 = 0.0011) was the difference in tumor sizes for the occurrence of Δ133p53α in cancer and normal tissue.

**Table 3:** Comparison of p53 isoforms according to clinicopathological features

Clinicopathological factor	p53 Isoforms																	
	p53 wt α			p53 wt β			p53 wt γ			p53 △40γ			p53△133α			p53△133β		
	Negative (%)	Positive (%)	*p *value	Negative (%)	Positive (%)	*p *value	Negative (%)	Positive (%)	*p *value	Negative (%)	Positive (%)	*p *value	Negative (%)	Positive (%)	*p *value	Negative (%)	Positive (%)	*p *value
Cancer versus	18 (33)	37 (67)	0.022	43 (78)	12 (22)	0.141	53 (86)	2 (4)	0.271	55 (100)	0 (0)	0.495	46 (84)	9 (16)	0.580	53 (49)	55 (51)	0.495
Normal	7 (13)	48 (87)		35 (64)	20 (36)		49 (89)	6 (11)		53 (96)	2 (4)		49 (89)	6 (11)		2 (100)	0	

Clinicopathological factor	p53 isoforms in cancer tissue																	
	p53 wt α			p53 wt β			p53 wt γ			p53 △40γ			p53△133 α			p53△133β		
	Negative (%)	Positive (%)	*p *value	Negative (%)	Positive (%)	*p *value	Negative (%)	Positive (%)	*p *value	Negative (%)	Positive (%)	*p *value	Negative (%)	Positive (%)	*p *value	Negative (%)	Positive (%)	*p *value
Metastatic (NanyM1) versus	2 (12)	15 (88)	1.000	8 (47)	9 (53)	0.130	14 (82)	3 (18)	0.359	17 (100)	0 (0)	1.000	16 (94)	1 (6)	0.654	16 (94)	1 (6)	0.527
Non-metastatic (N0M0)	5 (13)	33 (87)		27 (71)	11 (29)		35 (92)	3 (8)		36 (95)	2 (5)		33 (87)	5 (13)		37 (97)	1 (3)	
T1a	2 (13)	14 (87)	0.884	8 (50)	8(50)	0.372	16 (100)	0 (0)	0.269	15 (94)	1 (6)	0.162	12 (75)	4 (25)	0.347	15 (94)	1 (6)	0.976
T1b	3 (23)	10 (77)		10 (77)	3 (23)		12 (92)	1 (8)		13 (100)	0 (0)		11 (85)	2 (15)		13 (100)	0 (0)	
T2a	0 (0)	3 (100)		3 (100)	0(0)		3 (100)	0 (0)		2 (67)	1 (33)		3 (100)	0 (0)		3 (100)	0 (0)	
T2b	0 (0)	1 (100)		0 (0)	1 (100)		1 (100)	0.000		1 (100)	0 (0)		1 (100)	0 (0)		1 (100)	0 (0)	
T3a	2 (10)	17 (90)		12 (63)	7 (37)		14 (74)	5 (26)		19 (100)	0 (0)		19 (100)	0 (0)		18 (95)	1 (5)	
T3b	0 (0)	2 (100)		1 (50)	1 (50)		2 (100)	0 (0)		2 (100)	0 (0)		2 (100)	0 (0)		2 (100)	0 (0)	
T4	0 (0)	1 (100)		1 (100)	0 (0)		1 (100)	0 (0)		1 (100)	0 (0)		1 (100)	0 (0)		1 (100)	0 (0)	
Localized (T1 + T2) versus	5 (15)	28 (85)	0.689	21 (64)	12 (369	1.000	32 (97)	1 (3)	0.033	31 (94)	2 (6)	0.511	27 (82)	6 (18)	0.071	32 (97)	1 (3)	1.000
Locally advanced (T3 + T4)	2 (9)	20 (91)		14 (64)	8 (36)		17 (77)	5 (23)		22 (100)	0 (0)		22 (100)	0 (0)		21 (96)	1 (4)	
N0	7 (14)	45 (86)	1.000	32 (62)	20 (38)	0.293	47 (90)	5 (10)	0.298	50 (96)	2 (4)	1.000	46 (89)	6 (11)	1.000	50 (96)	2 (4)	1.000
N +	0 (0)	3 (100)		3 (100)	0 (0)		2 (67)	1 (33)		3 (100)	0 (0)		3 (100)	0 (0)		3 (100)	0 (0)	
M0	5 (13)	34 (87)	1.000	28 (72)	11 (28)	0.067	36 (92)	3 (8)	0.342	37 (95)	2 (5)	1.000	34 (87)	5 (13)	0.660	38 (97)	1 (3)	0.501
M1	2 (12)	14 (87)		7 (44)	9 (56)		13 (81)	3 (18)		16 (100)	0 (0)		15 (94)	1 (6)		15 (94)	1 (6)	
G1	1 (11)	8 (89)	0.735	3 (33)	6 (67)	0.221	9 (100)	0 (0)	0.596	8 (89)	1 (11)	0.549	6 (67)	3 (33)	0.127	9 (100)	0 (0)	0.572
G2	3 (11)	25 (89)		19 (68)	9 (32)		24 (86)	4 (14)		27 (96)	1 (4)		26 (93)	2 (7)		26 (93)	2 (7)	
G3	2 (13)	13 (87)		11 (73)	4 (27)		13 (87)	2 (13)		15 (100)	0 (0)		14 (93)	1 (7)		15 (100)	0 (0)	
G4	1 (33)	2 (67)		2 (67)	1 (33)		3 (100)	0 (0)		3 (100)	0 (0)		3 (100)	0 (0)		3 (100)	0.000	
Female versus	0 (0)	17 (100)	0.086	7 (41)	10 (59)	0.033	14 (82)	3 (18)	0.359	15 (88)	2 (12)	0.092	15 (88)	2 (12)	1.000	17 (100)	0 (0)	1.000
Male gender	7 (18)	31 (82)		28 (74)	10 (26)		35 (92)	3 (8)		38 (100)	0 (0)		34 (89)	4 (11)		36 (95)	2 (5)	
Mean tumor size (SEM) isoform																		
Yes	*n* = 7	6.69 (1.98)	0.94	*n* = 35	6.7 (0.72)	0.70	*n* = 49	6.38 (0.60)	0.33	*n* = 53	6.63 (0,56)	0.58	*n* = 49	6.95 (0.59)	0.001	*n* = 53	6.63 (0.57)	0.28
No	*n* = 48	6.52 (0.57)		*n* = 20	6.26 (0.87)		*n* = 6	7.87 (1.30)		*n* = 2	4.4 (2,9)		*n* = 6	3.27 (0.67)		*n* = 2	4.2 (1.3)	
Mean patient age (SEM) isoform																		
Yes	*n* = 7	64.43 (2.54)	0.25	*n* = 35	66.17 (1.5)	0.16	*n* = 49	67.8 (1.34)	0.42	*n* = 53	67.4 (1,27)	0.22	*n* = 49	66.9 (1.3)	0.16	*n* = 53	67.26 (1.26)	0.005
No	*n* = 48	67.96 (1.35)		*n* = 20	69.85 (2.07)		*n* = 6	65.17 (2.80)		*n* = 2	70.5 (1,5)		*n* = 6	72.5 (3.3)		*n* = 2	74 (1.00)	

Test statistics were done with Fisher’s exact or the χ^2^ test as appropriate for categorical variables, continues variables were compared with Student’s *t *test. Significant different p53 isoform expressions for p53 wtα in cancer versus normal tissue, for p53 wtβ in male and female gender, for p53 wtγ in localized versus advanced tumor stages, for p53∆133α in tumor sizes, and p53∆133β in mean patient ages. However, after correction for multiple comparisons only the difference in tumor sizes could be considered significant different for p53∆133α

## Discussion

The overexpression of p53 has been linked to poor prognosis in ccRCC and it was the aim of the current study to get more insight into the role of p53 role in ccRCC. p53 is the most frequently mutated gene in human cancer(Kastenhuber and Lowe 2017). For example, Giacomelli et al. (2018) have demonstrated that p53 mutants can be found in several cancer types including tumors of the intestine, the CNS, bladder, ovary, skin, liver, and lung. In clinical practice, the IHC pattern of p53 has been often interpreted as mutated pattern (null or diffuse) and wildtype (mosaic). In our study, only 12.6% would be wildtype (mosaic) and 87.4% would be mutated (mainly null). However, the current study has demonstrated that somatic mutations are a rare event when analyzing the TCGA data in ccRCC. Our findings are in line with previous findings of mutational analyses in ccRCC (Noon et al. 2011; Hakimi et al. 2013). For example, Sato et al. (2013) found in their analysis only in 3/106 patients a somatic mutation. Therefore, the common IHC staining pattern interpretation is not correct for ccRCC.

However, in a most recent publication of Motzer et al*.* mRCC tumors have been classified into seven molecular subgroups. In this analysis, *Tp53* mutations could be found in 107/836 (13%) of all and 84/701(12%) ccRCC tumor samples, respectively (suppl. Table 1 in Motzer et al. 2020). The slightly higher mutation frequency of *Tp53* in this series may be attributed to the fact that the cohort analyzed by Motzer et al*.* included samples that were gained from primary tumors of mRCC patients while previous analyses included numerous tumor samples of localized ccRCC patients (suppl. Table 1). Additionally, in the study of Motzer et al*.* numerous tumor samples of patients with ccRCC with sarcomatoid dedifferentiation and non-ccRCC were included. In both sarcomatoid and non-ccRCC tumors p53 mutations are a more frequent event than in pure ccRCCs.

The low mutation may underscores that p53 is not a significant driver mutation in ccRCC. Albers et al. found that combined inactivation of the von Hippel Lindau (VHL) gene and *Tp53* induces simple cysts which have some precursor features of ccRCC but inactivation of VHL and p53 was not sufficient enough to induce ccRCC development (Albers et al. 2013). Even though, additional inactivation of the retinoblastoma gene (Rb) is able to induce ccRCC like tumors in *VHL*^Δ/Δ^,*Tp53*^Δ/Δ^,*Rb*^Δ/Δ^ mice, these tumors do not demonstrate metastatic potential (Giacomelli et al. 2018; Harlander et al. 2017).

On the other hand, previous work has suggested that p53 protein levels are higher in tumors of patients with advanced tumor stages and higher levels of p53 are associated with poor prognosis in ccRCC (Noon et al. 2010). Our in in-vitro experiments have demonstrated an upregulation of p53 and pp53 after radiation of several ccRCC cell lines, but the up-regulation of p53 does not affect migration, proliferation and transcriptional activity. This finding may explains in part the resistance of ccRCC against conventional radiation dosages and cytotoxic agents. Additionally, it demonstrates that the mechanisms of the activation of p53 expression are intact while the transition to transcriptional activity is inhibited in ccRCC.

We have seen an univariable association of p53 protein levels in the TCGA data as well as our TMA analysis. Therefore, in ccRCC may exists the paradox situation that the *wildtype* tumor suppressor p53 is overexpressed but is unable to induce its genuine functions including apoptosis, senescence and cell cycle arrest. In the current study, we have functionally demonstrated that p53 could be induced in ccRCC cell lines. Furthermore, p53 is also activated as shown by its increase in phosphorylation. Although p53 was overexpressed and activated in the current study, there was no inhibition of proliferation migration, and no activation of p53 transcriptional activity after irradiation of ccRCC cell lines in our experiments. Harlander et al. (2017) have demonstrated that numerous genes that encode proteins which regulate p53 transcriptional activity, demonstrate gains and losses which indicate their inactivation in ccRCC. For example, *PBRM1* loss is a frequent event in ccRCC and *PBRM1* loss decreases p53 mediated transcriptional regulation of *CDKN1A* (p21) (Cai et al. 2019; Giacomelli et al. 2018). Furthermore, the most common mutational event in ccRCC is the inactivation of the VHL gene (Nickerson et al. 2008). VHL interacts with KAT5 (Tip60) which acetylates and thereby activates p53 (Roe et al. 2011; Giacomelli et al. 2018). These findings support the hypothesis that p53 is although activated functionally inhibited in ccRCC. In line with these genetic implications, the current study was unable to demonstrate an increase in p53 transcriptional activity after irradiation as demonstrated in our findings of a p53 reporter assay.

Another plausible explanation for functional inhibition of p53 in ccRCC could be the impact of hypoxia on the function of p53. It has been discussed that hypoxia results in p53 accumulation. Under conditions of severe hypoxia p53 may be able to destabilize and inhibit HIF-1α. In turn, both transcription factors compete for transcriptional co-factors like p300. Since HIF-1α and HIF-2α are of utmost importance for the tumor biology of ccRCC, interactions of hypoxia inducible factors in general and HIF-1α in particular could provide additional explanations for a functional inhibition of p53 (Schmid et al. 2004).

p53 isoforms were investigated in ccRCC cell lines and tumor samples to find plausible explanations for mechanisms that may lead to inhibition of p53 functions in ccRCC. Isoforms of p53 have been linked to cancer in general and are upregulated in those cancer entities that demonstrate a low p53 mutation rate (reviewed in Vieler and Sanyal 2018). Furthermore, upregulation of p53 isoforms has been linked to tumor types like breast, colorectal or multiple myeloma cancer (reviewed in Vieler and Sanyal 2018). In the current study, a patient cohort of 55 patients with ccRCC was screened for p53 isoforms. The only significant result that we have found were smaller tumor sizes in tumors harboring Δ133p53α. This isoform has been described as a pro-survival factor and an inhibitor of senescence, apoptosis, and p53 transcriptional activity (reviewed in (Vieler and Sanyal 2018)). However, in the current analyses, the opposite could be demonstrated: tumor samples demonstrating Δ133p53α had smaller tumor sizes and not advanced sizes as could be expected if Δ133p53α would be an inhibitor of senescence, apoptosis, and p53 transcriptional activity. Additionally, none of the six patients harboring Δ133p53α in their tumor samples as compared with 11/49 (22%) of patients without Δ133p53α had died at the time of data cutoff. The difference is with respect to the small sample size not significant, but it may emphasize the unique role of p53 in ccRCC. We have investigated different p53 isoforms as one possible inhibition factor but were unable to demonstrate a link between p53 isoforms and advanced tumor stages. There are numerous other publications that describe mechanisms of p53 inhibition. With respect to ccRCC biology, possible further inhibition mechanisms could include e.g. interactions with the NF-κB pathway (Gurova et al. 2005), PBRM1 (Macher-Goeppinger et al. 2015) and members of the thioredoxin family (Ueno et al. 1999).

The current study has limitations that need to be considered when interpreting the results. The TMA and the p53 isoforms cohorts investigated in this study have limited follow up data. In both cohorts less than 50% of patients have died from RCC or other reasons and thus, the interpretation of the survival outcome could be limited. Furthermore, the number of metastatic ccRCC in these two cohorts is compared to patient cohorts of previous studies that have demonstrate p53 as a poor prognostic factor rather low (Klatte et al. 2009). This may be an explanation why p53 could only be validated as a poor prognostic factor in univariable analysis. Lastly, larger sample sizes could also make some trends clearer. For example, the association of Δ133p53α with less advanced tumor and probably better survival outcome is may be more evident in a larger patient cohort with longer follow up data. The demonstrated induction of p53 and pp53 expression after radiation of the ccRCC cell lines were not measured over a time period of e.g. 24, 48, 72, 96 h etc. Therefore, it could be that the observed effect of p53 and pp53 induction does not last exceptionally long.

Collectively, the current study provides additional indications that the usually present wildtype tumor suppressor gene p53 has a loss of function in ccRCC. This finding seems to be a unique situation in ccRCC. To date the mechanisms that are responsible for this inhibition still must be defined but in the light of many ccRCCs that are resistant to current standard of care therapies, this functional inhibition may be an attractive target for another treatment strategy in future.

## Supplementary Information

Below is the link to the electronic supplementary material.

Supplementary file1 (DOCX 4073 kb)

## Data Availability

Data are mentioned in the manuscript are freely available in the TCGA found at http://cancergenome.nih.gov or will be provided upon request from the corresponding author.
