# Supplementary material for: p53 is functionally inhibited in clear cell renal cell carcinoma (ccRCC): a mechanistic and correlative investigation into genetic and molecular characteristics
Source: J Cancer Res Clin Oncol. 2021 Sep 9;147(12):3565–76. doi: 10.1007/s00432-021-03786-1 (PMC8557161; doi:10.1007/s00432-021-03786-1)
Supplement: Supplementary file 1 — Supplementary file1 (DOCX 4073 kb) [file 432_2021_3786_MOESM1_ESM.docx]

p53 is functionally inhibited in clear cell renal cell carcinoma (ccRCC): A mechanistic and correlative investigation into genetic and molecular characteristics.

### Karoline Diesing*1 Silvia Ribback*4, Stefan Winter*3, Manuela Gellert2, Antonia M. Oster2, Viktoria Stühler7, Eva Gläser2, Frank Adler5, Christoph Hartwig5, Markus Scharpf6, Jens Bedke7, Martin Burchardt1, Matthias Schwab3,8, Christopher H. Lillig2,π, Nils Kroeger1,2,π

From the Department of Urology, University Medicine Greifswald, Germany (1); the Institute of Medical Biochemistry and Molecular Biology, University Medicine Greifswald, Germany(2); the Dr. Margarete Fischer-Bosch Institute of Clinical Pharmacology, Stuttgart, and the University of Tübingen, Tübingen, Germany (3); the Institute of Pathology, University Medicine Greifswald, Germany (4); the Institute of Radiation Oncology, University Medicine Greifswald, Germany (5), the Institute of Pathology, University of Tübingen, Germany (6); the Department of Urology, University of Tübingen, Germany (7); Departments of Clinical Pharmacology, Pharmacy and Biochemistry, University of Tübingen, Tübingen, Germany (8)

# Corresponding author: Nils Kroeger, University Medicine, Department of Urology, Ferdinand-Sauerbruch-Straße, DE-17475 Greifswald, Germany, phone: +49 3834 865979, fax: +49 3834 865978, e-mail: [md.nkroeger@gmail.com](mailto:md.nkroeger@gmail.com)

# Supplementary Materials

**Figures:**

**Supplementary Figure 1:**


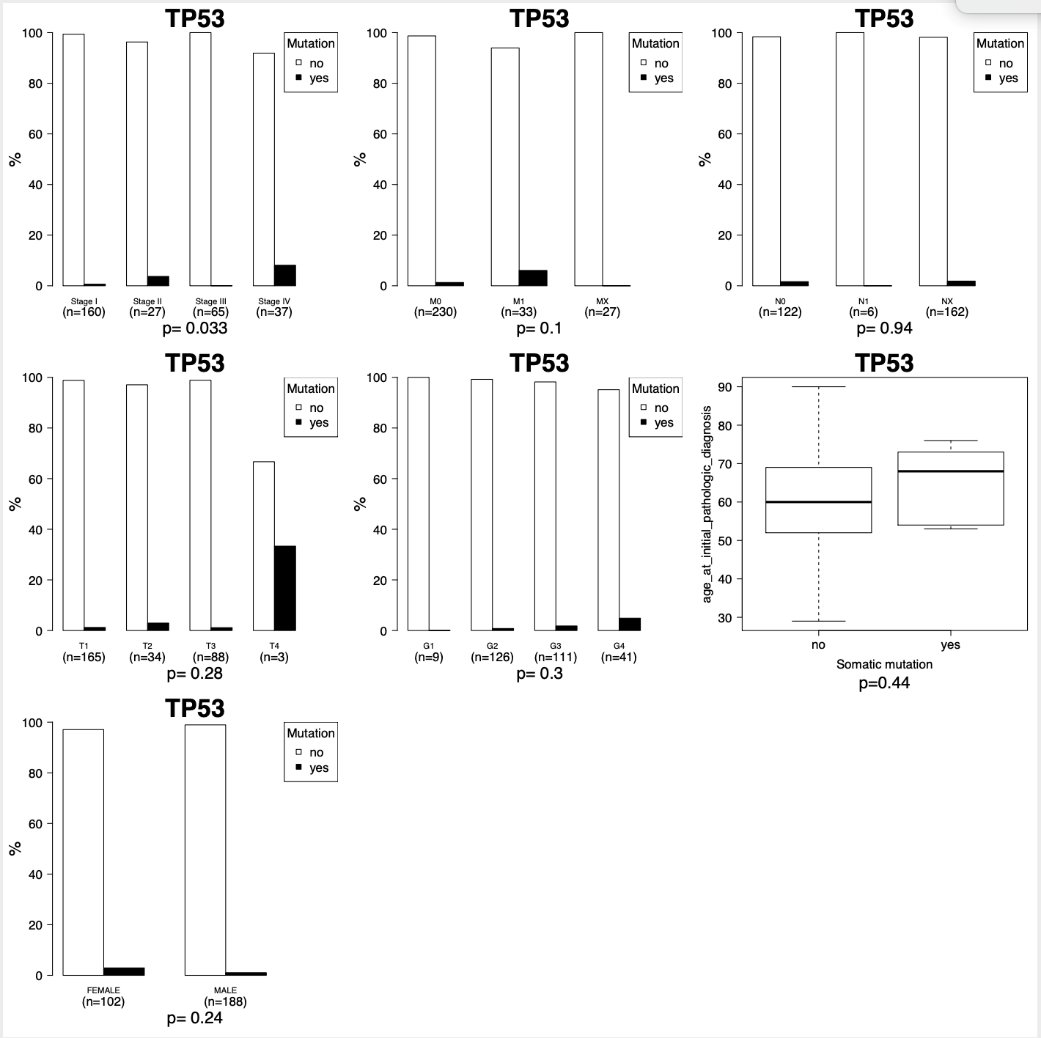


**Suppl. Figure 1** demonstrates the association of somatic p53 mutations with tumor stages and clinicopathological features in the TCGA in ccRCC. Somatic p53 mutations could only be found in 5 of 290 analyzed ccRCC tumor samples. There was no clear trend for an association of somatic p53 mutations with advanced tumor stages.

**Supplementary Figure 2:**

| 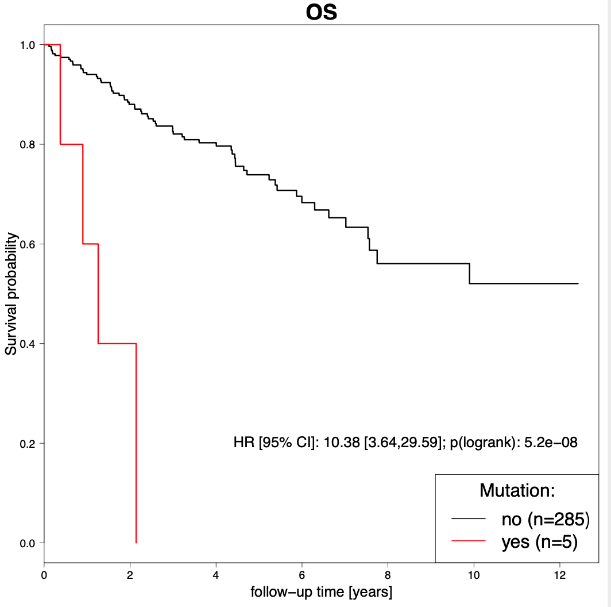  **Suppl Figure 2 A** | 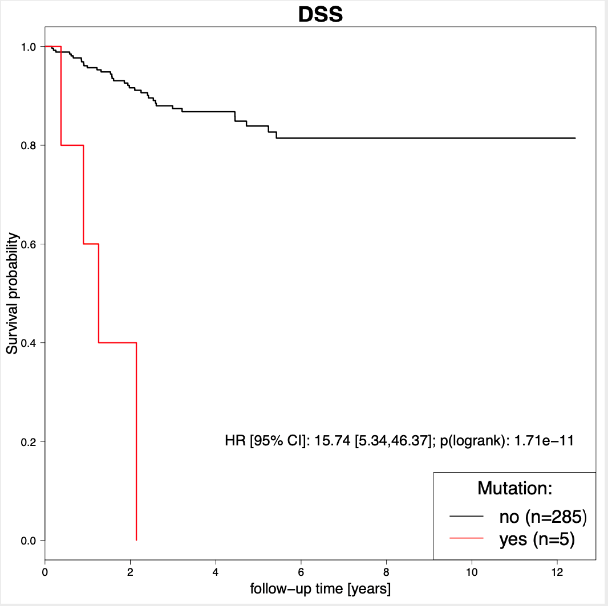  **Suppl Figure 2 B** | 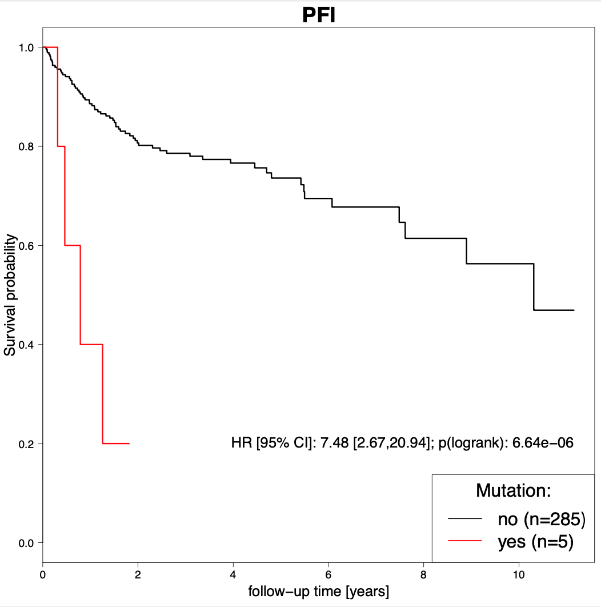  **Suppl Figure 2 C** |
| --- | --- | --- |

**Suppl Figure 2** demonstrates the association of somatic mutations with overall, cancer specific and progression-free survival in the TCGA in ccRCC. Somatic mutations could only be found in 5 of 290 patients but these patients had significantly worse survival outcome when compared to patients whose tumor samples did not have somatic p53 mutations.

**Supplementary Figure 3:**

| 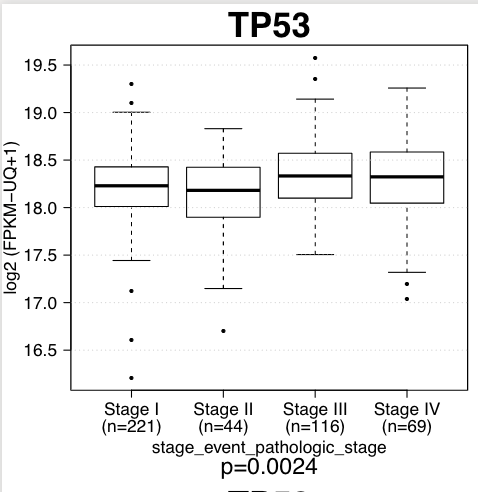 A | 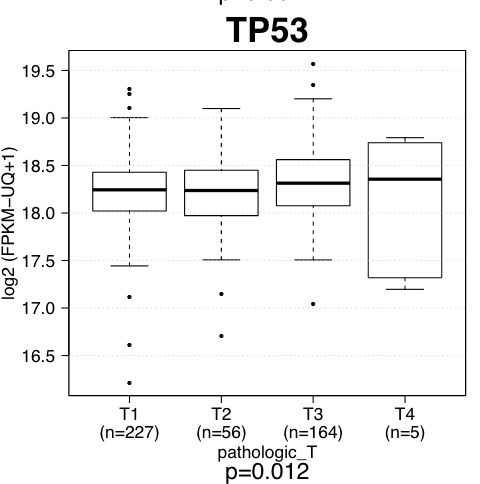 B | 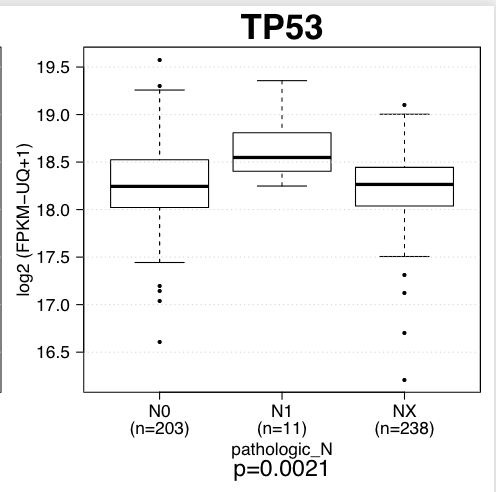 C |
| --- | --- | --- |
| 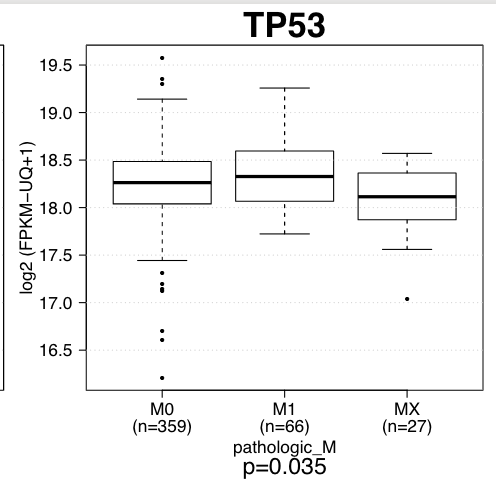 D | 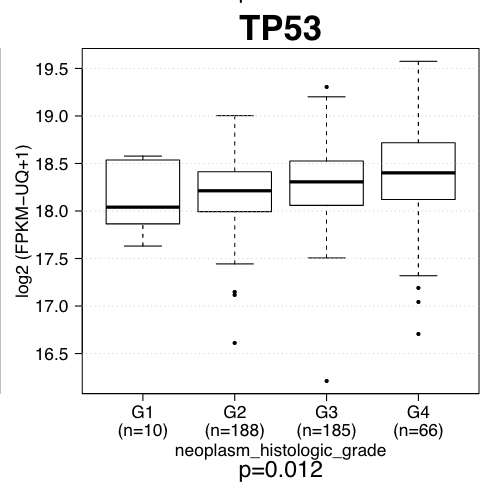 E | **Suppl. Figure 3** A-E demonstrates the associaten of p53 mRNA levels with clinical stages and pathological tumor features. p53 was marginally higher expressed in tumor sampleas with higher Tstages, Fuhrmann grades, distant, and lymph node metastases. |

**Supplementary Figure 4:**

| 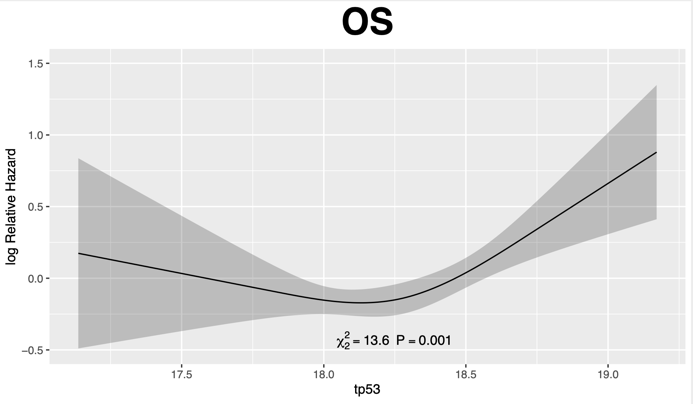 | 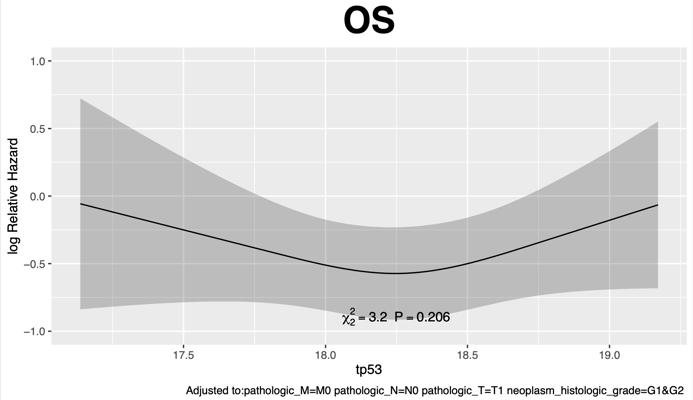 |
| --- | --- |
| 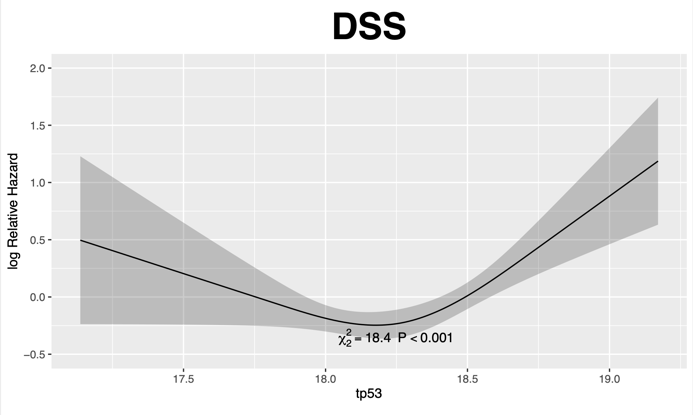 | 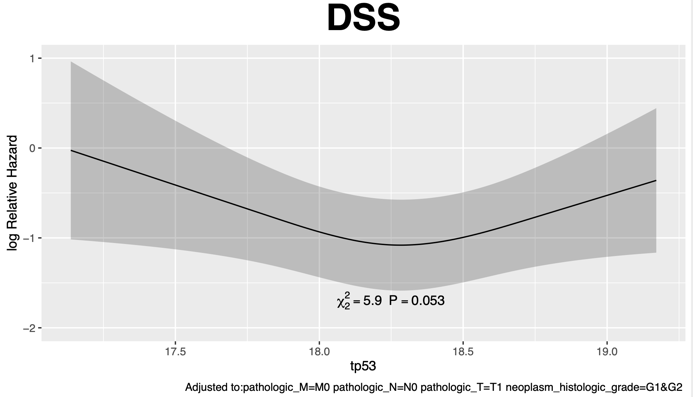 |
| 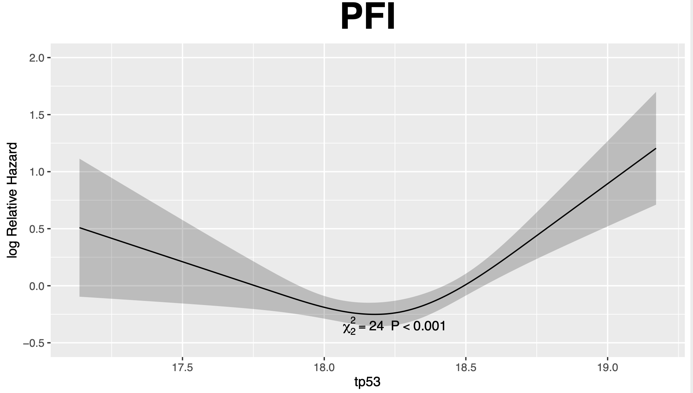 | 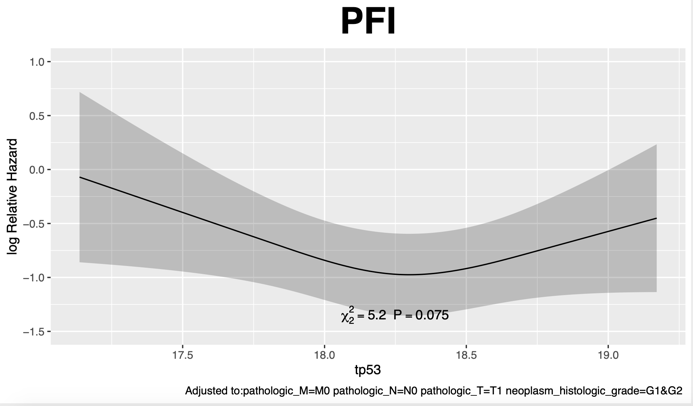 |

**Supplementary Figure 4** demonstrates univariable association of p53 mRNA levels with survival on the left side and the multivariable association on the right side. The results show that there was no monotonous association between p53 RNA levels (RNA seq data) and survival outcome in all endpoints. Best survival outcome was observed with intermediate expression levels.

**Supplementary Figure 5**

| 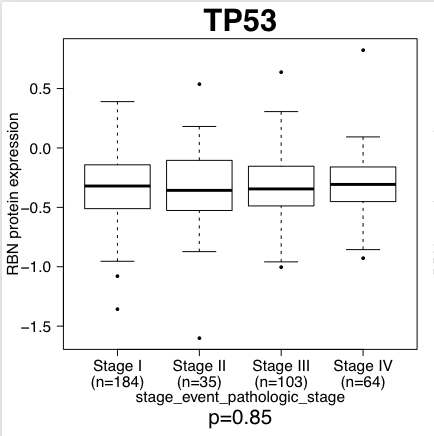 | 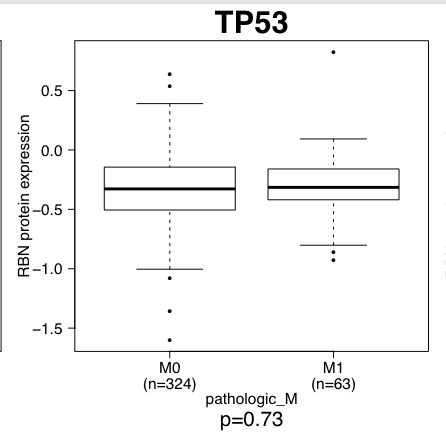 |
| --- | --- |
| 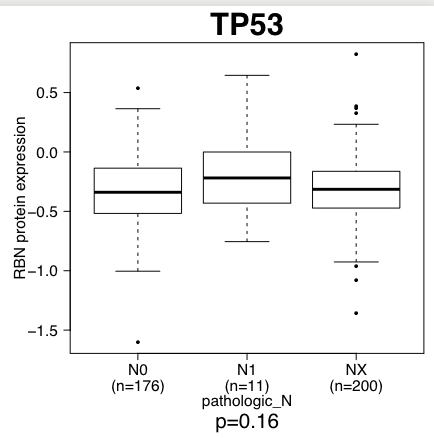 | 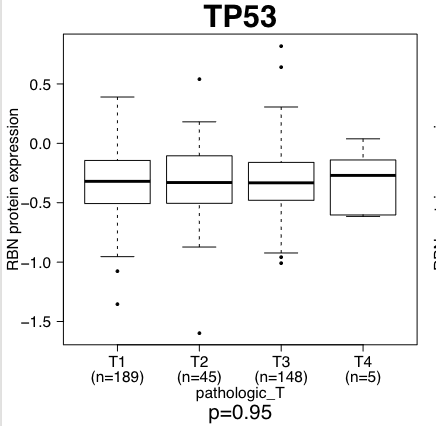 |
| 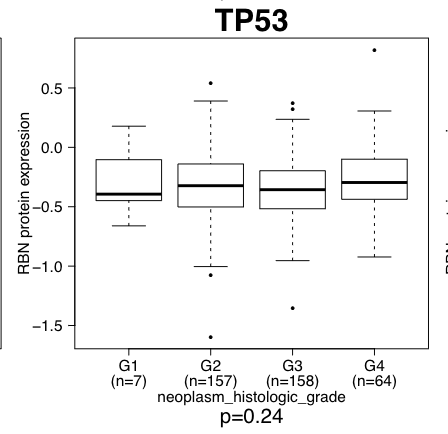 | **Suppl. Figure 5** demonstrates the association of p53 protein levels with clinicopathological features. There was no significant association of p53 levels with clinicopathological features. |

**Supplementary Figure 6**


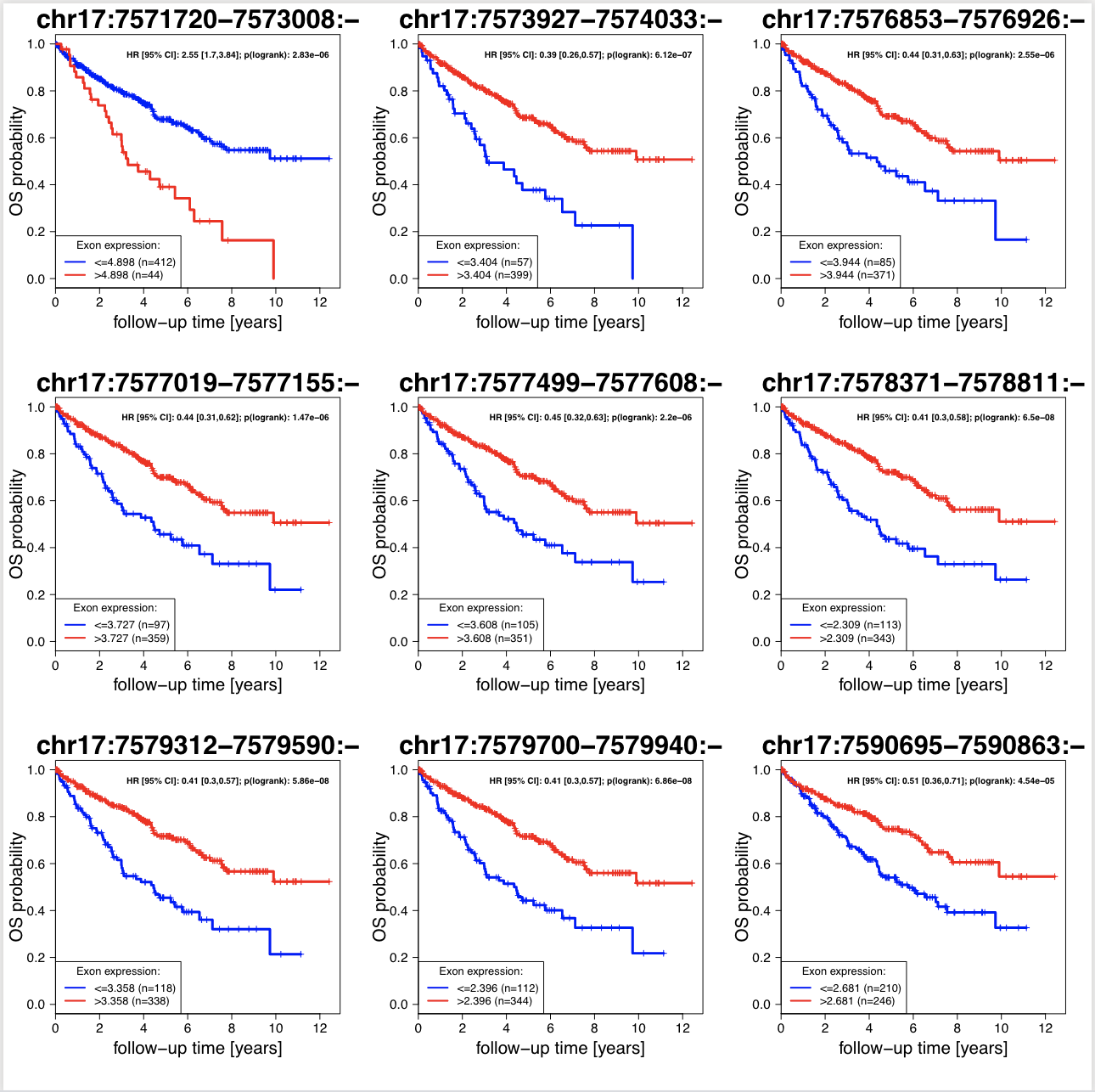


**Supplementary Figure 7:**


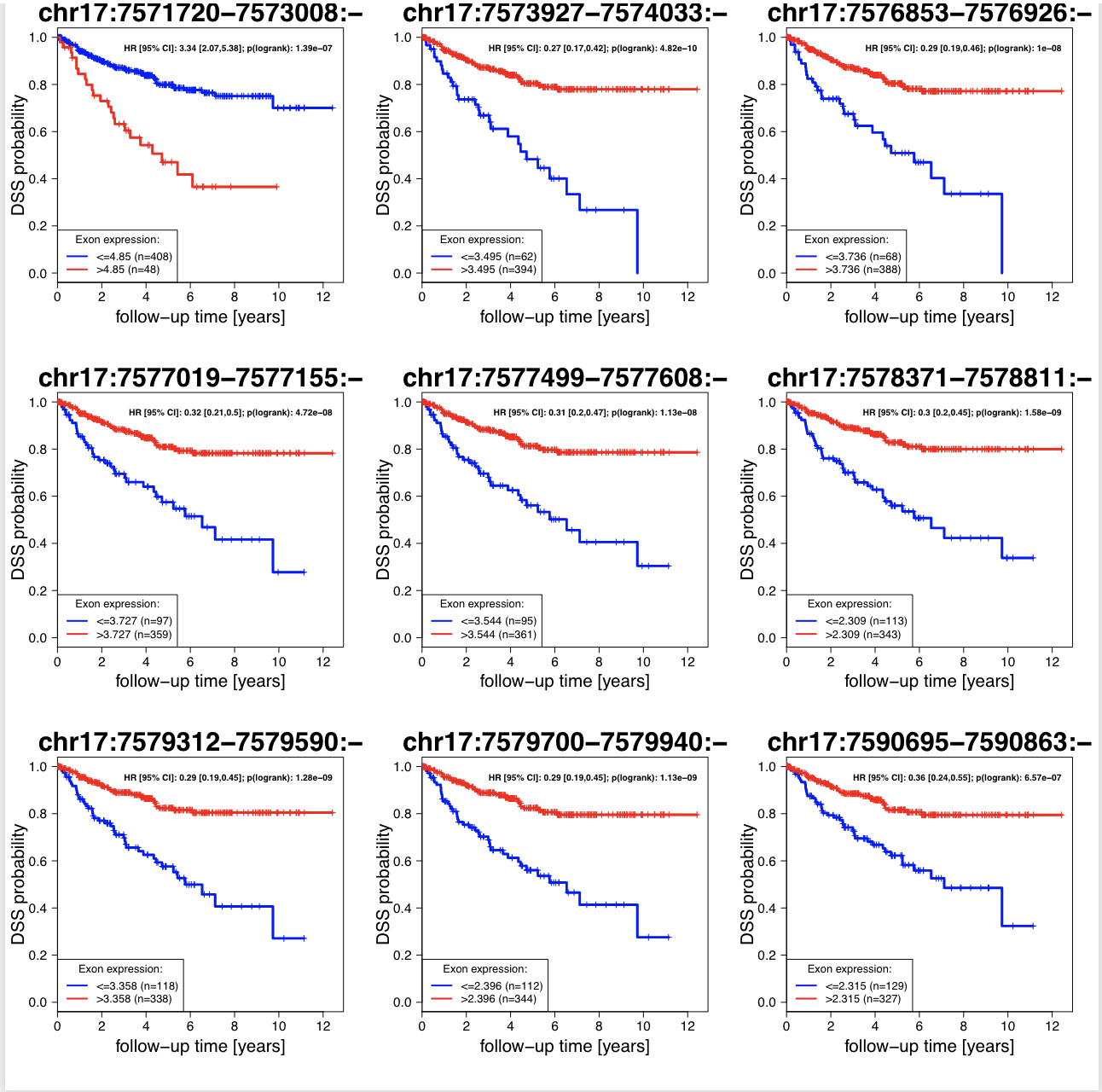


**Supplementary Figure 8**


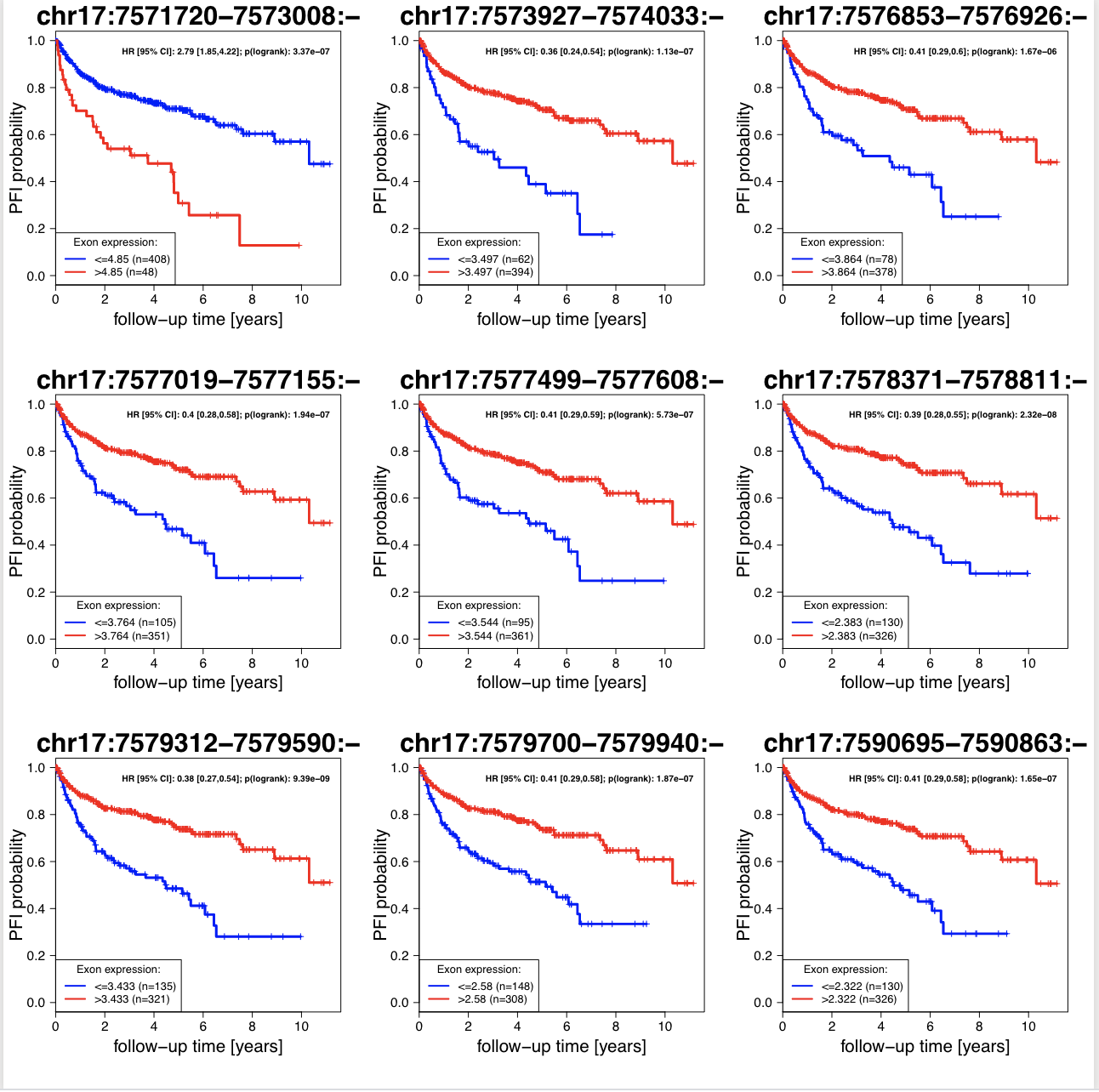


**Supplementary Figure 9**

| 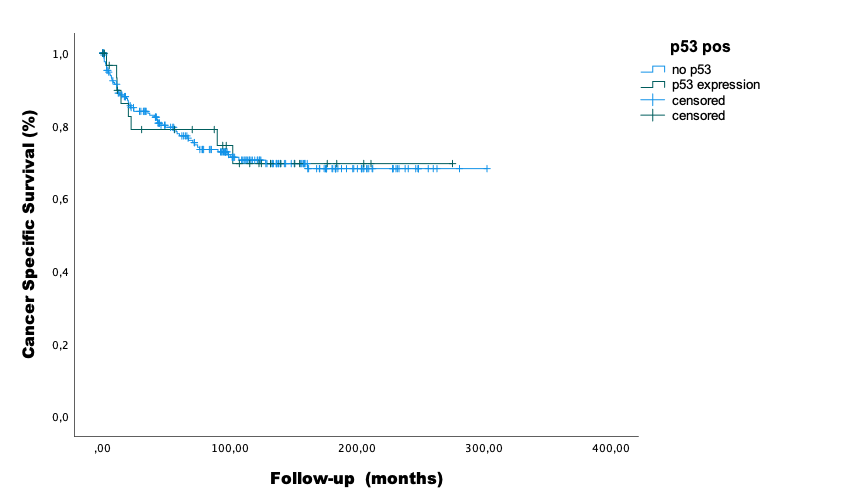  **Supplementary Figure 9A** The estimated mean survival times were 204,0 (95% CI: 161,9 – 246,1) vs. 220,6 (95% CI: 202,5 – 238,6); p=0,943 in patients with tumors that had vs. did not have p53 expression in their primary tumor specimens. |
| --- |
| 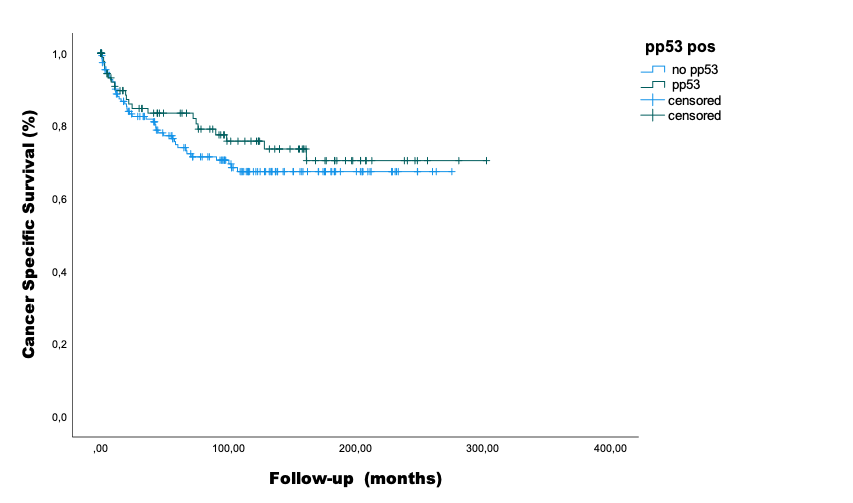  **Supplementary Figure 9B** The estimated mean survival times were 229,8 (95% CI: 202,9 – 256,8) vs. 197,8 (95% CI: 178,7 – 217,02); p=0,381 in patients with tumors that had vs. did not have pp53 expression in their primary tumor specimens |

**Supplementary Figure 10.**

|  | **H&E** | **p53** | **pp53** |
| --- | --- | --- | --- |
| **positive** | **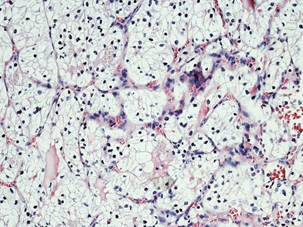** | **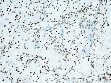** | **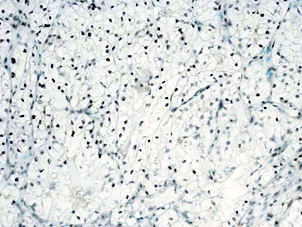** |
| **negative** | **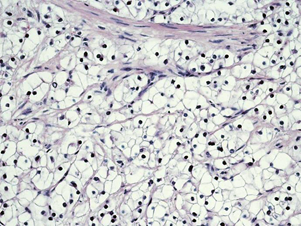** | **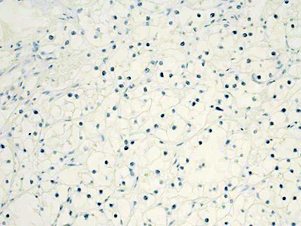** | **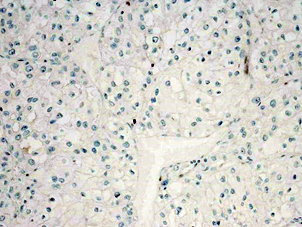** |

Length of the lower edge 1mm/0,5mm.

**Supplementary Tables:**

**Supplementary Table 1**

**Clinicopathological features of the TCGA cohorts:**

| **Characteristics of patients/primary tumors** | **levels/summary statistics** | **n=459 KIRC tumors** | |
| --- | --- | --- | --- |
|  |  | **No.** | **%** |
| **Sex** | male | 293 | 63,8 |
|  | female | 166 | 36,2 |
| **Age (years) at diagnosis of primary RCC** | median (range) | 61 (29-90) | |
| **T** | 1 | 230 | 50,1 |
|  | 2 | 56 | 12,2 |
|  | 3 | 168 | 36,6 |
|  | 4 | 5 | 1,1 |
| **N** | 0 | 205 | 44,7 |
|  | 1 | 12 | 2,6 |
|  | X | 242 | 52,7 |
| **M** | 0 | 364 | 79,3 |
|  | 1 | 68 | 14,8 |
|  | X | 27 | 5,9 |
| **G^a^** | 1 | 10 | 2,2 |
|  | 2 | 192 | 41,8 |
|  | 3 | 186 | 40,5 |
|  | 4 | 68 | 14,8 |
|  | X | 1 | 0,2 |
| **Stage^a^** | I | 224 | 48,8 |
|  | II | 44 | 9,6 |
|  | III | 118 | 25,7 |
|  | IV | 71 | 15,5 |
| **Follow-up time (years)** | median (range) | 3.6 (0-12.4) | |
| **Vital status** | alive | 310 | 67,5 |
|  | dead | 149 | 32,5 |
| **Cancer-related death^a^** | no | 360 | 78,4 |
|  | yes | 89 | 19,4 |
| **Progression‐free-intervall** | no | 321 | 70 |
|  | yes | 138 | 30 |
| ^a^Percentages do not sum up to 100% due to missing values. | |  |  |

**Clinical characteristics of the analyzed TCGA KIRC cohort (n=459). In this analysis, patients with none-ccRCC and neoadjuvant treatment have been excluded according to Chen et al. (Chen, F., Zhang, Y., Senbabaoglu, Y. et al.: Multilevel Genomics-Based Taxonomy of Renal Cell Carcinoma. Cell Rep, 14: 2476, 2016). Available data sets have been different for the individual analyses.**

**Supplementary Table 2**:

| **Clinicopathological parameters of the cohort analyzed for p53 isoforms.** | | |
| --- | --- | --- |
| **Feature** | **Parameter** | **Patients** |
| **Age** | Median (IQR) | 70 (59–74) |
| **Follow-up** | Median (IQR) | 11.2 (0.23–19.1) |
| **Gender (%)** | Male  Female | 38 (69.1)  17 (30.9) |
| **T-Stage (%)** | T1a  T1b  T2a  T2b  T3a  T3b  T3c  T4 | 16 (29.1)  13 (23.6)  3 (5.5)  1 (1.8)  19 (34.5)  2 (3.6)  0  1 (1.8) |
| **N-Stage (%)** | N0  N1 | 52 (94.5)  3 (5.5) |
| **M-Stage (%)** | M0  M1 | 39 (70.9)  16 (29.1) |
| **Fuhrman Grading (%)** | G1  G2  G3  G4 | 9 (16.4)  28 (50.9)  15 (27.3)  3 (5.5) |
| **Sarcomatoid Features (%)** | Yes  No | 7 (12.7)  48 (87.3) |
| **Cancer-specific death (%)** |  | 22 (20.0) |

**Supplementary Table 3** Antibodies used for immunohistochemistry

| **Protein** | **product # / [reference]** | **company / source** | **antigen unmasking** | **antibody dilution** |
| --- | --- | --- | --- | --- |
| Phospho p53 | Ab38497 | abcam | boiling, pH 6 | 1:100 |
| p53 | M7001 | DAKO | boiling, pH 6 | 1:50 |

Formalin-fixed, paraffin-embedded sections of five µm thickness were stained for p53 and phosphorylated p53 (Table 3) in an automated immunostainer (Benchmark XT, Roche Ventana) using a DAB (diaminobenzidine) kit.

## Methods Tissue Micro Array

For the tissue micro arrays (TMA) study, core tissue biopsies (0.6 mm in diameter) were taken from selected morphologically representative regions of paraffin-embedded renal tumor and precisely arrayed using a custom-built instrument. Each block was evaluated from an experienced pathologist before it was included in the study. Then 4 µm thick sections of the resulting tumor tissue microarray block were transferred to glass slides The process has been described previously in detail [1, 2]. The staining was evaluated by experienced genitourinary pathologist (S.R. and M.S.). The intensity was counted on a 0-3 scale (0=negative, 1=weakly positive, 2=moderately positive, 3=strongly positive), and percentage of positively stained target cells (range 0-100% positive) staining at each intensity.

## Methods of In-vitro Experiments

**Cell culture and treatment**

Four Human RCC cell lines (786-O, A-498, CaKi-1, RCC4) and one adult kidney cell line (RC-124) were purchased from CLS Celllines Service GmbH, Eppelheim, Germany, and one cell line (RCC4) from Public Health England. The cell line 786-O was cultured in RPMI medium, A-498 and CaKi-1 in MEM Eagle supplemented with 1% sodium pyruvate (PAN Biotech, Aidenbach, Germany), RCC4 in DMEM supplemented with 1% sodium pyruvate and 1% glutamine (PAN Biotech) and RC-124 was cultured in McCoy´s Medium. All media were obtained from PAN Biotech and were supplemented additionally with 1% Penicillin and Streptomycin (PAN Biotech) and 10% Fetal Calf Serum (FCS, Invitrogen, Darmstadt, Germany). Every cell line was routinely analyzed for mycoplasm contamination.

The molecular characteristics have been described in the literature as follows:

- - CAKI-1 ( p53 wild type), ccRCC [3, 4]
  - RCC4 (p53 wt), ccRCC [3-5]
  - 786-O (p53 mut), ccRCC [3, 4]
  - A-498 ( p53 wt), ccRCC [3, 6]
  - RC-124 (established from non-malignat kidney tissue according to CLS Cellines Service GmbH)

It was the aim to include cell lines with wildtype p53, mutated p53 and a cell line that was established from a non-malignant part of the kidney.

**Migration assay**

Migration activity of RCC and adult kidney cells were investigated by scratch assay. RCC cells were cultured on 24-well plates in complete medium until 100% confluence was reached. A scratch was caused with a 200 µl tip. Cell migration was observed in ZEISS Observer Z.1 (Carl ZEISS AG, Oberkochen, Germany) microscope with maintained normal growth conditions and microscopic pictures were taken directly after wounding and every 30 minutes in a period of 2 days. Wound healing was evaluated by ZEISS ZEN microscope Software.

**Reporter Gene Assay**

Activity of functional p53 was investigated by Dual Luciferase Assay (Promega, Madison, WI, USA) after transient transfection of cells with plasmids from Cignal p53 Reporter Assay (Qiagen, Venlo, Netherlands). RCC cells were transfected by Lipofectamine 2000 (Invitrogen, Darmstadt, Germany). For transfection with Lipofectamine 2000 cells were cultured in 96-wells for 1-2 days and media were replaced with medium without any supplements. The amount of plasmid DNA for RCC cell lines A-498, 789-O, CaKi-1 and RCC4 was 0.3 µg per well, for adult kidney cell line RC-124 0.4 µg per well. Procedure occured according to manufacturer´s instructions with a ratio of 1 µg DNA : 1.5 µl Lipofectamine 2000.

After transient transfection with a mixture of p53-inducible *firefly* vector and constitutively expressed *renilla* vector p53 expression was investigated by Dual Luciferase Assay following the manufacturer´s instructions and measured at Infinite M200 Pro (Tecan Group LTD, Männedorf, Schweiz). After incubation with reagents PLB and LARII, given by manufacturer, luminescence of *firefly* luciferase was measured with an integration time of 10 seconds. Adding of Stop&Glo led to a stop reaction and *renilla* signal was detected with 10 seconds integration time. For quantification signals of *renilla* luciferase and negative control was subtracted from *firefly* signal. Analysis of p53 activity via reporter assay happened 5 d after irradiation.

Plasmids for positive and negative control were also provided and used. Positive controls were constitutively expressed vectors with *renilla* and *firefly* luciferases and Green Fluorescent Protein. A negative control contained a non-inducible *firefly* luciferase and a constitutively expressed *renilla* vector.

**Irradiation of cell lines**

Currently, standard of care therapies of metastatic RCC include tyrosine kinase inhibitors (TKI) and immune checkpoint inhibitors (CPI). RCC is known to be resistant against irradiation and conventional cytotoxic chemotherapies. The role of p53 in resistant mechanisms of RCC against irradiation is currently not well characterized. From a molecular basis, it seems to be rather unlikely that TKIs or CPIs are p53 inductors. On the other hand, it is very well known that irradiation could induce p53 expression [7].

Therefore, radiation of RCC cell lines was used in order to stimulate the pro-apoptotic function of p53. Characterization of RCC cells happened before and after irradiation with X-rays. Cells were proliferated for 48 h (786-O, CaKi-1, RCC-4) or 72 h (RC-124, A-498) at normal growth conditions and then irradiated with 2 Gy. Controls underwent the same handling as the treated cells except irradiation. Fractions of 2 Gray are clinically relevant radiation dosages to several tumor entities and were therefore chosen for irradiation of the cells. RCC is known to be resistant against radiation therapy but radiation in high dosages is increasingly recognized as a treatment option in both localized and metastatic RCC [8, 9].

**Cell proliferation (CASY and Trypanblau)**

Cell proliferation was detected in CASY Cell Counter + Analyser System Model TT (Roche Diagnostics GmbH, Basel, Schweiz). 100 µl of diluted cell suspension was mixed in 10 ml CASY ton (154 mM NaCl, 0,1 mM EDTA in A. bidest). Cell amount per ml was calculated by division of 4 from detected cell amount. Proliferation was measured every 24 h in a range of 0 to 168 h after irradiation.

Vitality was tested by trypan blue dye. Trypan blue was mixed with PBS in a ratio of 1:10 and this mixture was used 1:2 in cell suspension and examined in microscope for blue, dead cells. Clear cells maintained alive and vital.

**Westernblot analysis**

Proteins were detected 2 and 8 h after irradiation. Therefore, RCC cells were mechanically detached in DPBS (PAN Biotech), suspension was centrifuged for 5 min at 3000 U/min and the supernatant was discarded. Cell pellets were suspended in homemade lysis buffer containing 33.5 mM Tris (pH 7.5), 3.5 mM EDTA (pH 8.0), 100 mM NaCl, 6.7 mM K2HPO4, 6.7% v/v Glycerol, 0.67% Triton X-100, 0.035% v/v SDS, 1 mM sodium vanadate, 20 mM sodium fluoride, 20 mM glycerol-2-phosphate, 0.1 mM PMSF, 20% Complete Mini EDTA free (Roche Diagnostics) and ultrasonated. Especially for detection of hypoxic factors lysis buffer was added by 200 µM DFO and coated with liquid nitrogen. Total protein concentration was determined by conventional Bradford assay. Subsequently, proteins were size fractionated by sodium dodecylsulfate polyacrylamide gel electrophoresis using 5% stacking and 10% running gel. 100 µg protein was added each lane of gel and electrophoresis was started at 80V for 10 min and was running afterwards at 150V for about 90 min. Gels were transferred in a semi-dry Western Blot on nitrocellulosis membranes using Trans-Blot SD semi-dry transfer cell (Bio-Rad, München, Germany) with 0,06 mA each membrane. Protein transfer was verified through Ponceau S-Dying, membranes were blocked for 1 h in blocking solution (Carl Roth) and incubated over night at 4°C with monoclonal primary antibodies against p53 (1:10,000; BD Biosciences, Heidelberg, Germany), phospho-p53 (1:1,000; Cell Signaling, Danvers, USA), HIF-1α (1:200, Cell Signaling), HIF-2α (1:2,000; Novo Biologicals, Littleton, USA), PARP (1:10,000; Cell Signaling) and against GAPDH (1:10,000, Cell Signaling) as reference. Subsequently, membrane was incubated for 1 h with horseradish peroxidase-linked secondary antibody and developed using the ECL method. Chemoluminescence signals were detected using a CCD camera in ChemiDoc XRS+ and quantificated with Image Lab TM Software 3.0.

**PCR analysis of p53 isoforms.**

**mRNA isolation from human tissue samples**

Tumor and control samples of 55 patients, treated for clear cell renal cell carcinoma (RCC) with open or laparoscopic radical or partial nephrectomy between 2012-2015, from the Department of Urology, University Medicine Greifswald were analyzed, approved by the local review board (IRB protocol #BB 81a/12). All samples were immediately frozen in liquid nitrogen and stored at -80°C until further use. Clinicopathological features included gender, age, the 7th edition of AJCC/UICC tumor, node, and metastasis classification (TNM) 2010, Fuhrman grade, tumor size, sarcomatoid features, and the cancer specific survival time (CSS). Two pathologists (Silvia Ribback and Frank Dombrowski, chairman, Institute of Pathology, University Greifswald, Germany) staged and graded all tumors at the initial surgery and also confirmed the purity of the tissue included in this study.

RNA extraction was performed using 100 µl peqGOLD TriFast (Peqlab, Erlangen, Germany) per 10 mg tissue, the TissueLyser II system, and 5 mm stainless steel beads (Qiagen, Hilden, Germany) according to the manufacturer's instructions. Final RNA concentration was determined using the NanoDrop 2000c. The oligodT18 primer and 1 µg of RNA were used to prepare 30 µl of cDNA using the RevertAid First Strand cDNA Synthesis Kit (Thermo Fisher Scientific) according to the manufacturers protocol. All PCRs were performed using Taq Polymerase (Thermo Fisher Scientific). Unless otherwise indicated, 20 µl of the final reaction mixture contained 1.5 mmol∙L-1 MgCl2, 5% DMSO, 0.5 µmol∙L-1 each primer, 0.2 µmol∙L-1 dNTPs, 0.5 µl cDNA, and 1 U∙µL-1 Taq polymerase. Two types of PCR were performed. In the standard PCR, an initial denaturation step of 2 minutes was followed by 30 seconds of denaturation. After 40 seconds of annealing (temperatures see table XY), elongation ran at 72°C (specific elongation times see suppl. table 3). Denaturation, annealing, and elongation were repeated in 35 to 40 cycles, followed by a final elongation step. In the second type of PCR, the initial 10-15 cycles were performed at a different annealing temperature, as indicated in table XY. After the initial cycles the annealing temperature was adjusted to the target specific temperature either directly or in case of p53 Δ133β the temperature was adjusted using a gradient with 0.5°C decrease per cycle. All PCR products were visualised using 1% agarose gels in TAE buffer (40 mmol µmol∙L-1 Tris, 20 mmol µmol∙L-1 acetic acid, 1 mmol µmol∙L-1 EDTA, pH 8.5) and RedSafe (iNtRON Biotechnology) and the ChemidocTM XRS system. Specific PCR products were confirmed by sequencing (Microsynth Seqlab). The occurrence of each p53 isoform was categorised (yes = 1, no = 0) and Fisher’s exact test or the Chi-Square test were applied to compare differences in clinicopathological features. Combinations of different p53 isoforms were also analysed for their correlation with clinicopathological features. The IBM software SPSS V.24 was applied for all analyses and a significance level of p<0.05 was considered significant

**Supplementary Table 4** PCR Primers and PCR conditions used for evaluation of PCR transcript variants

| Control | | Primer sequence | | annealing temperature [°C] | elongation time [s] | cycles | protocol modification |
| --- | --- | --- | --- | --- | --- | --- | --- |
| GAPDH | | fwd. | 5’-CAAGGTCATCCATGACAACTTTG-3’ | 55 | 40 | 35 |  |
|  |  | rev. | 5’-GTCCACCACCCTGTTGCTGTAG-3’ |  |  |  |  |
| p53 isoforms | |  | |  |  |  |  |
| mRNA variant | Protein |  | |  |  |  |  |
| 1, 2 | WTα | fwd. | 5’-CAGTCAGATCCTAGCGTC-3’ | 55 | 80 | 40 |  |
|  |  | rev. | 5’-CTCATTCAGCTCTCGGAAC-3’ |  |  |  |  |
| 3 | WTβ | fwd. | 5’-TCAGATCCTAGCGTCGAG-3’ | 55 | 80 | 40 |  |
|  |  | rev. | 5’-AAGCTGGTCTGGTCCTGA-3’ |  |  |  |  |
| 4 | WTγ | fwd. | 5’-TCCTGAAAACAACGTTCTGTCC-3’ | initial: 55°C/15 cycles  final: 62°C/ 25 cycles | 60 | 40 |  |
|  |  | rev. | 5’-AGTCAAGTAGCATCTGAAGGGTGA-3’ |  |  |  |  |
| 4 | Δ40γ | fwd. | 5’-CAGAAAACCTACCAGGGCAGCTAC-3’ | initial: 55°C/15 cycles  final: 62°C/ 25 cycles | 60 | 40 | 3.5 mM MgCl_2_ |
|  |  | rev. | 5’-AGTCAAGTAGCATCTGAAGGGTGA-3’ |  |  |  |  |
| 5 | Δ133α | fwd. | 5’-GTCTCCTTCCTCTTCCTAC-3’ | initial: 58°C/10 cycles  final: 55°C/ 30 cycles | 45 | 40 |  |
|  |  | rev. | 5’-CTCATTCAGCTCTCGGAAC-3’ |  |  |  |  |
| 6 | Δ133β | fwd. | 5’-GTCTCCTTCCTCTTCCTAC-3’ | initial: 63°C/15 cycles, gradient reducing temperature by 0.5°C per cycle  final: 55°C/ 25 cycles | 45 | 40 | 3% DMSO |
|  |  | rev. | 5’-AAGCTGGTCTGGTCCTGA-3’ |  |  |  |  |

[1] Kim HL, Seligson D, Liu X, Janzen N, Bui MH, Yu H, et al. Using protein expressions to predict survival in clear cell renal carcinoma. Clin Cancer Res. 2004;10:5464-71.

[2] Kononen J, Bubendorf L, Kallioniemi A, Barlund M, Schraml P, Leighton S, et al. Tissue microarrays for high-throughput molecular profiling of tumor specimens. Nature medicine. 1998;4:844-7.

[3] (IARC) IAfRoC. IARC TP53 database. <http://p53.iarc.fr/:> International Agency for Research on Cancer; 2013.

[4] Tsao CC, Corn PG. MDM-2 antagonists induce p53-dependent cell cycle arrest but not cell death in renal cancer cell lines. Cancer Biol Ther. 2011;10:1315-25.

[5] Khan MN, Bhattacharyya T, Andrikopoulos P, Esteban MA, Barod R, Connor T, et al. Factor inhibiting HIF (FIH-1) promotes renal cancer cell survival by protecting cells from HIF-1alpha-mediated apoptosis. Br J Cancer. 2011;104:1151-9.

[6] Kucejova B, Pena-Llopis S, Yamasaki T, Sivanand S, Tran TA, Alexander S, et al. Interplay between pVHL and mTORC1 pathways in clear-cell renal cell carcinoma. Molecular cancer research : MCR. 2011;9:1255-65.

[7] Lavin MF, Gueven N. The complexity of p53 stabilization and activation. Cell death and differentiation. 2006;13:941-50.

[8] Siva S BM, Wood S, Shaw M, Loi S, Sandhu SK, Tran B, Azad A, Lewin JH, Cuff K, Neha N, Colyer D, Neeson PJ, Liu H, Chander S, Moon D, Goad J, Murphy DG, Lawrentschuk N, Pryor D, MacCallum P. Stereotactic radiotherapy and pembrolizumab for oligometastatic renal tumors: The RAPPORT trial. J Clin Oncol 2021;39:suppl 6 abstract 277.

[9] Staehler M, Bader M, Schlenker B, Casuscelli J, Karl A, Roosen A, et al. Single fraction radiosurgery for the treatment of renal tumors. J Urol. 2015;193:771-5.
